# Bridging the gap from mechanism to clinic: a translational perspective on taVNS for gastrointestinal disorders

**DOI:** 10.3389/fnins.2026.1820521

**Published:** 2026-06-22

**Authors:** Jian Cui, Jingxue Zhao, Shuanglin Zhou

**Affiliations:** 1 Department of General Surgery, Beijing Hospital, National Center of Gerontology, Beijing, China; 2 National Clinical Research Center for Gerontology, Beijing, China; 3 The Key Laboratory of Geriatrics of NHC, Beijing, China; 4 Institute of Geriatric Medicine, Chinese Academy of Medical Sciences, Beijing, China; 5 Guang’anmen Hospital, China Academy of Chinese Medical Sciences, Beijing, China

**Keywords:** brain–gut axis, gastrointestinal disorders, neuromodulation, transcutaneous auricular vagus nerve stimulation, vagus nerve

## Abstract

In recent years, multiple clinical and translational studies have investigated the application and mechanisms of transcutaneous auricular vagus nerve stimulation (taVNS) in gastrointestinal disorders (GIDs), with consideration given to pathophysiology, stimulation parameters, and patient-specific factors. In this review, we systematically synthesize recent evidence from clinical trials and preclinical models published in leading gastroenterology and neurology journals. Our focus is on taVNS-mediated modulation of the brain–gut axis, particularly its role in improving autonomic balance, reducing visceral sensitivity, and attenuating inflammatory responses, with the aim of enhancing therapeutic outcomes in functional and inflammatory GIDs. There is a need to optimize stimulation protocols through mechanistic insights and to promote the use of this non-invasive, well-tolerated neuromodulation approach. These advances are essential for expanding taVNS accessibility in clinical practice, especially for patients with refractory symptoms, comorbid psychological conditions, and in settings where conventional treatments are limited or contraindicated. Personalized taVNS strategies and biomarker-guided dosing represent emerging trends in neuromodulation therapy. However, standardized protocols and predictive models have yet to be established for widespread clinical implementation.

## 1 Introduction

Gastrointestinal Disorders (GIDs) refer to a range of conditions affecting the esophagus, stomach, intestines, and related digestive organs. Common clinical manifestations include abdominal pain, bloating, constipation, diarrhea, and indigestion (Duffy et al., 2023). Globally, GIDs such as functional dyspepsia (FD) and constipation-predominant irritable bowel syndrome (IBS-C) account for a significant proportion of outpatient cases, with a prevalence exceeding 40% (Black et al., 2020; Di Rosa et al., 2023). Furthermore, inflammatory GIDs like inflammatory bowel disease (IBD), along with iatrogenic issues such as opioid-induced constipation (OIC) and postoperative ileus, further exacerbate the disease burden and healthcare costs (Bruner et al., 2023; Squeo et al., 2024; Wang Z. et al., 2025; Fan et al., 2025). The pathophysiological mechanisms of these disorders are complex, involving multifactorial interactions including genetic susceptibility, environmental factors, and lifestyle influences (Mukhtar et al., 2019; Bedell and Friedlander, 2022). GIDs have become a significant global public health challenge, not only resulting in high prevalence and substantial healthcare burdens but also markedly reducing patients’ quality of life (Person and Keefer, 2021). Despite advances in medical interventions and preventive measures, the overall burden of GIDs continues to rise (Sperber et al., 2021). Therefore, exploring effective treatment strategies is crucial for improving patient outcomes and enhancing quality of life.

Among various treatment strategies, pharmacotherapy remains the primary clinical approach for managing GIDs. Traditional medications such as acid suppressants, prokinetics, laxatives, and anti-inflammatory drugs can alleviate certain symptoms but face limitations in efficacy, safety, or long-term use (Drossman, 2016; Ford et al., 2014). For instance, some patients exhibit poor response to existing treatments or encounter issues like side effects and drug dependency (Fikree and Byrne, 2021). Consequently, identifying and developing novel, highly effective, and safe therapeutic agents represents a critical direction for current research.

## 2 The vagus nerve serves as a vital bridge regulating the brain-gut axis

As a complex and dynamic bidirectional communication network between the nervous system and the gastrointestinal tract, the brain-gut axis theory serves as a key foundation for understanding the mechanisms underlying GIDs and certain inflammatory conditions. GIDs such as FD and irritable bowel syndrome (IBS) are frequently accompanied by gastrointestinal dysmotility symptoms, visceral hypersensitivity, and central sensitization. These characteristics are closely associated with dysregulation of brain-gut signaling pathways (Gracie et al., 2019; Chen et al., 2022). Furthermore, in organic diseases like IBD, brain-gut interactions also participate in the neuro-regulation of immune modulation and inflammatory responses (Gracie et al., 2019). This suggests that dysfunction of the brain-gut axis not only represents a core mechanism in functional disorders but also plays a significant role in the development and persistence of certain inflammatory diseases (Margolis et al., 2021).

**FIGURE 1:**
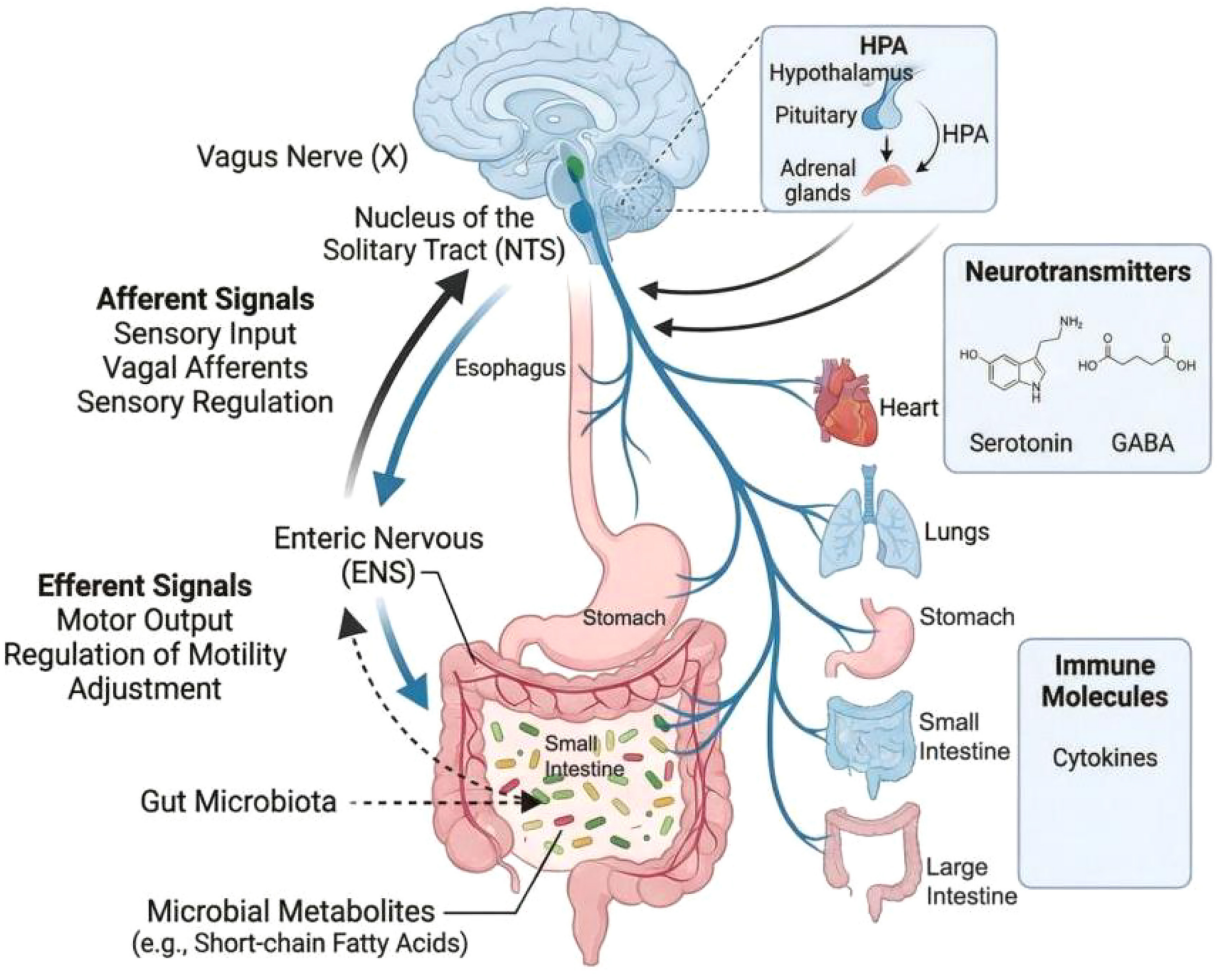
Schematic of vagus nerve-mediated bidirectional brain-gut axis communication. This diagram illustrates the vagus nerve as the core conduit for afferent and efferent signals between the central nervous system and gut. It also depicts crosstalk with the HPA axis, neurotransmitters, and gut microbiota, highlighting the axis’s neuro-immune-endocrine integration. Blue, neural structures; Pink, gut structures; Green, parasympathetic function; Arrows, signal pathways.

The vagus nerve constitutes the most critical anatomical and functional link in the brain-gut axis (Yuan and Silberstein, 2016). As the longest parasympathetic nerve in the body, it contains approximately 80% afferent fibers and 20% efferent fibers. Its afferent fibers transmit mechanical, chemical, and immune signals from the gastrointestinal tract via the ganglion to the solitary tract nucleus in the brainstem. While its efferent fibers act on the enteric nervous system and effector cells by releasing acetylcholine, directly regulating gastrointestinal motility, secretion, blood flow, and mucosal barrier function (Ma et al., 2025; Wang Y. et al., 2025). Importantly, beyond the canonical, splenic cholinergic anti-inflammatory pathway (CAP) that has been implicated in systemic inflammatory and autoimmune disorders, the vagus nerve modulates intestinal immune homeostasis and inflammatory responses primarily through a non-canonical, intestinal CAP. In this pathway, vagal (and sacral) parasympathetic efferents project directly onto the intestinal wall, where acetylcholine acts on α7 nicotinic acetylcholine receptors expressed on resident muscularis macrophages to suppress NF-κB signaling and pro-inflammatory cytokine release independently of the spleen (Matteoli and Boeckxstaens, 2016; de Araujo and de Lartigue, 2020). This plays a crucial physiological role in maintaining mucosal barrier integrity and alleviating intestinal inflammation (Bonaz et al., 2017). Additionally, the vagus nerve participates in regulating visceral sensory transmission, with its functional state closely linked to the perception of symptoms such as abdominal pain and bloating in functional GIDs (Person and Keefer, 2021; Figure 1).

Given the vagus nerve’s central regulatory role in the brain-gut axis, targeting its function has emerged as a cutting-edge therapeutic strategy for GIDs. Methods such as vagus nerve electrical stimulation, biofeedback training, neuromodulatory drugs, or behavioral therapies can modulate brain-gut signaling, restore gastrointestinal motility and secretory balance, reduce visceral sensitivity, and suppress excessive inflammatory responses (Yan et al., 2024; Décarie-Spain et al., 2024). Research indicates that vagal dysfunction—manifesting as reduced vagal tone and impaired brain-gut communication—is a common feature across multiple GIDs, including FD, IBS, and chronic abdominal pain (Zhu et al., 2021; Liu et al., 2024b; Sabo et al., 2021). Consequently, modulating vagal activity represents a rational therapeutic approach for restoring gastrointestinal homeostasis, holding significant promise for clinical translation (Browning et al., 2017).

## 3 The origin and development of transcutaneous auricular vagus nerve stimulation

The origins of transcutaneous auricular vagus nerve stimulation (taVNS) trace back to extensive research on vagus nerve stimulation (VNS), a technology initially approved by the U.S. Food and Drug Administration (FDA) for treating drug-resistant epilepsy and depression. Early VNS required surgical implantation of a cuff electrode around the vagus nerve in the neck. While demonstrating efficacy, this approach carried risks including surgical complications, infections, and device-related side effects (Mena-Chamorro et al., 2025). These limitations spurred researchers to explore non-invasive alternatives, driving the development of transcutaneous cervical vagus nerve stimulation (tcVNS) and taVNS (Bucksot et al., 2020). taVNS specifically targets the auricular branch of the vagus nerve (ABVN), which is distributed in the outer ear, particularly concentrated in the concha and tragus regions (Sagui et al., 2024; Kreisberg et al., 2021). Its anatomical basis stems from neurophysiological studies demonstrating that electrical or tactile stimulation of these auricular regions activates vagal afferent fibers, projecting signals to key brainstem nuclei such as the nucleus tractus solitarius (NTS) and locus coeruleus (LC) (Badran et al., 2019; Zhang Q. et al., 2024). Early studies in the early 21st century laid a crucial foundation for taVNS, confirming its ability to regulate autonomic function by modulating heart rate variability (HRV) and enhancing parasympathetic activity (Lee et al., 2023; Sagui et al., 2024). The non-invasive nature of taVNS and its mild side effects (such as localized skin irritation) have facilitated its rapid adoption in clinical and research settings (Giraudier et al., 2025; Kim et al., 2022).

The evolution of taVNS has been marked by technical refinements in electrode design and stimulation parameters. Computational modeling has played a pivotal role in optimizing electrode placement, aiming to selectively activate the auricular branch of the vagus nerve while minimizing off-target effects (Kreisberg et al., 2021). For instance, multiple controlled studies indicate that stimulation of the concha boat region elicits more pronounced vagal modulation responses compared to areas like the tragus, making it a more ideal target site (Kreisberg et al., 2021; Badran et al., 2019). Furthermore, personalized and standardized stimulation protocols have further enhanced the tolerability and efficacy of taVNS (Badran et al., 2019; Giraudier et al., 2025).

In clinical applications, the scope of taVNS has expanded beyond its initial use in treating epilepsy and depression to encompass stroke rehabilitation, cognitive function improvement, and intervention in inflammatory diseases (Zhang Q. et al., 2024; Capone et al., 2025). Its therapeutic potential primarily stems from the technology’s multifaceted regulatory effects on neuroplasticity, anti-inflammatory pathways, and autonomic balance (Sagui et al., 2024; Bucksot et al., 2020). Recent years have witnessed deepening research: on one hand, wearable taVNS devices suitable for long-term use have been developed; on the other, its combined application with other neuromodulation techniques like transcranial magnetic stimulation (TMS) is being explored. These advances collectively propel a deeper understanding of taVNS’s mechanisms of action (Capone et al., 2025).

In summary, the emergence of taVNS not only addresses the limitations of traditional invasive VNS but also benefits from the interdisciplinary convergence of neuroanatomy, bioengineering, and clinical neuroscience. Its development aligns with the broader trend in neuromodulation toward non-invasive and personalized approaches. Current research focuses on optimizing stimulation parameters, establishing treatment standards, and expanding its clinical indications (Kreisberg et al., 2021; Badran et al., 2019; Austelle et al., 2022; Zhang Q. et al., 2024). Notably, while preliminary research outcomes have been achieved in recent years regarding taVNS applications for GIDs, systematic reviews in this area remain limited (Shi et al., 2021; Liu et al., 2025). Therefore, this paper aims to provide reference and insights for future research and clinical practice of taVNS in gastrointestinal diseases by reviewing existing clinical evidence and mechanisms of action.

## 4 Methods

### 4.1 Search strategy

A systematic search was conducted in the PubMed, Embase, Web of Science, and Cochrane Library databases for clinical studies published from the inception of each database until December 10, 2025, aiming to identify literature investigating taVNS for gastrointestinal diseases. The search strategy combined Medical Subject Headings (MeSH) with free-text keywords, focusing primarily on terms and their combinations such as “transcutaneous auricular vagus nerve stimulation,” “transcutaneous vagus nerve stimulation,” “auricular vagus nerve stimulation,” “gastrointestinal diseases,” “functional dyspepsia,” “irritable bowel syndrome,” “constipation,” “colitis,” “inflammatory bowel disease,” “postoperative ileus,” “opioid-induced constipation,” “gastroparesis,” “visceral sensitivity,” “brain-gut axis,” and “abdominal pain.” Literature types included single-arm trials, randomized controlled trials (RCTs), case reports, and non-randomized controlled trials.

### 4.2 Research screening process

The research screening process comprised two stages: First, the review panel (JC and JXZ) independently conducted an initial review of the titles and abstracts of all retrieved records. If inclusion could not be determined based on the title and abstract alone, the records proceeded to the second stage, where full texts were obtained and reviewed for further evaluation. Any discrepancies arising during screening were resolved through internal discussion or by inviting a third-party arbitrator. To enhance screening efficiency and accuracy, retrieved literature records were first imported into EndNote software (Analytics Inc., Philadelphia, United States) for automated deduplication and preliminary title/abstract screening. Records passing this initial screening were subsequently imported into Zotero 5.0 (Digital Scholar LLC, Vienna, United States) for full-text review and final confirmation. This screening process imposed no restrictions on publication date or language.

Inclusion criteria were as follows: (1) original research articles; (2) clinical trials involving patients with FGIDs; (3) intervention involving taVNS or tVNS or auricular vagus nerve stimulation; (4) study design being randomized controlled trial, single-arm taVNS trial, case report and non-randomized controlled trials; and (5) studies published in English.

**FIGURE 2:**
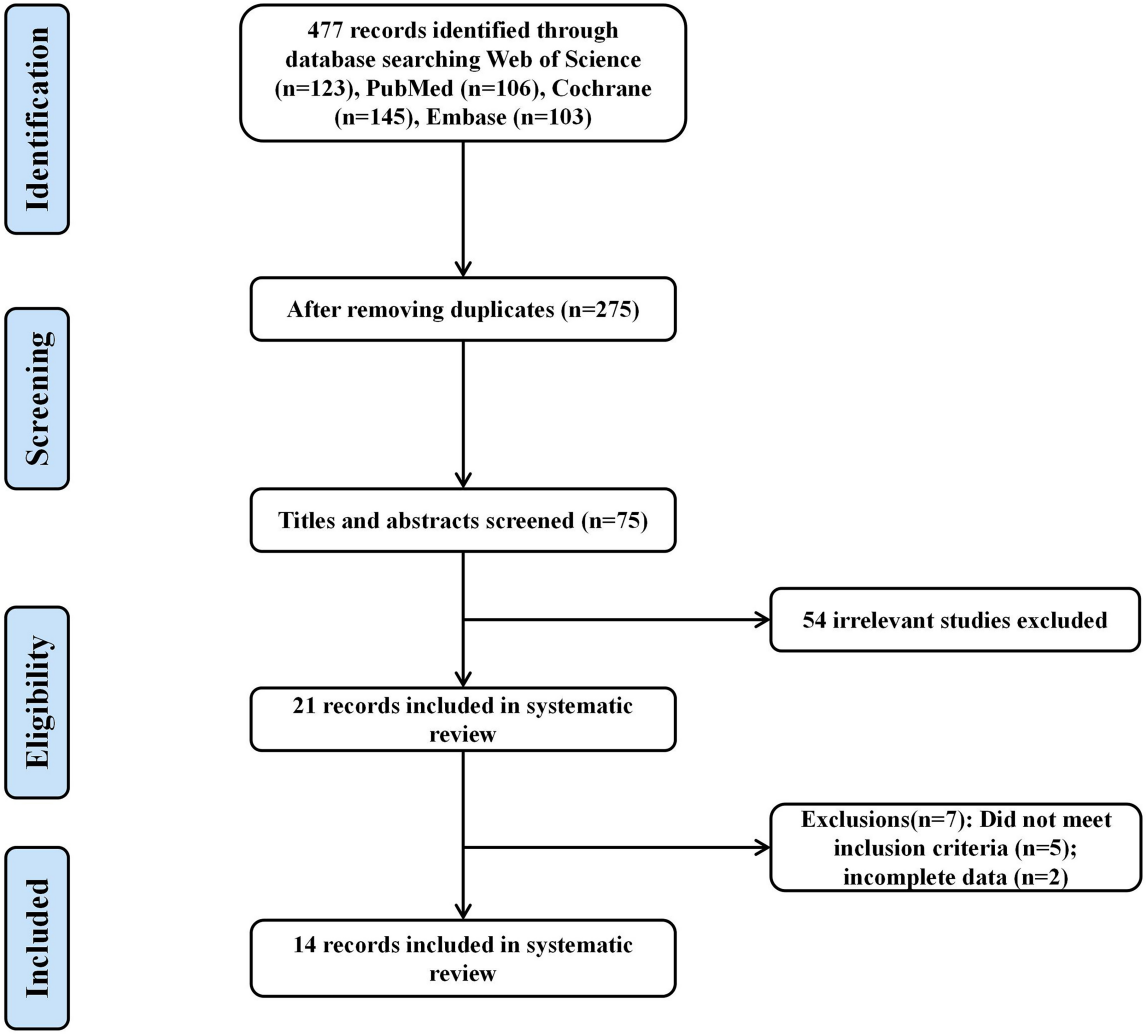
Flow chart of study selection.

Exclusion criteria: (1) Non-original research types, including meta-analyses, systematic reviews, case reports, commentary articles, letters to the editor, and conference abstracts; (2) Non-electrical stimulation interventions for the trigeminal nerve (e.g., acupuncture or auricular acupressure); (3) Failure to report relevant outcome measures; (4) Studies where data could not be obtained or calculated despite contacting authors; (5) Studies not focused on gastrointestinal diseases (Figure 2).

### 4.3 Data extraction

Data extraction and quality assessment were performed independently by two reviewers, SLZ and JXZ. For studies lacking relevant outcome information, we contacted the original authors via email to obtain unpublished details. Extracted information primarily covered the following aspects: (1) First author and publication year; (2) Patient population characteristics, such as gastrointestinal disease type, and sample size, including control and intervention groups; (3) Stimulation location; (4) Intervention protocol details, including stimulation parameters such as frequency, intensity, pulse width, and duration; (5) Primary outcome measures (Table 1).

**TABLE 1:** Clinical studies of taVNS in gastrointestinal disorders.

References	Population	Study design	Group	Stimulation site	taVNS parameter	Main findings
Zhu et al. (2021)	FD patients (*n* = 36)	Randomized controlled trial	taVNS vs. Sham-ES	taVNS:bilateral auricular cymba conchae Sham-ES: lateral to the arm wrist	Frequency: 25 Hz Pulse width: 0.5 ms Intensity: 0.5–1.5 mA Duration: 2 weeks	Improved gastric accommodation, increased percentage of normal gastric slow waves, enhanced vagal activity, reduced dyspeptic symptoms, anxiety, and depression scores.
Dong et al. (2021)	FD patients (*n* = 90)	Randomized controlled trial	taVNS vs. tnVNS	taVNS: left cavum conchae tnVNS: left scapha	Frequency: 30 Hz Intensity: 4–8 mA Duration: 4 weeks	taVNS can sustainably improve core symptoms, anxiety and depression levels, and quality of life in patients with FD, with therapeutic effects persisting for up to 12 weeks post-treatment.
Shi et al. (2024)	FD patients (*n* = 300)	Multicenter, Randomized controlled trial	10 Hz taVNS vs. 25 Hz taVNS vs. Sham-taVNS	taVNS: left tragus Sham: left earlobe	Frequency: 10 Hz (V10) or 25 Hz (V25) Pulse Width: 500 μs Intensity: 0.5–1.5 mA Duration: 4 weeks	Both taVNS frequencies significantly improved FD symptoms and quality of life vs. sham, with sustained efficacy and a favorable safety profile.
Li et al. (2025)	FD patients with sleep disturbance (*n* = 54)	Randomized controlled trial	taVNS vs. tnVNS	taVNS: bilateral auricular cymba concha tnVNS: bilateral auricular scapha	Frequency: 25 Hz Pulse width: 0.5 ms Intensity: 2–10 mA Duration: 4 weeks	Improved FD symptoms, sleep, mood, and quality of life, enhanced gastric function and melatonin, correlated with vagal activity, and was safe.
Steidel et al. (2021)	Healthy individuals (*n* = 57)	Randomized, double-blind trial	HF taVNS (25 Hz) vs. LF taVNS (1 Hz)	Left cymba conchae	HF: 25 Hz, 250 μs pulse width, biphasic LF: 1 Hz, 250 μs pulse width, biphasic Duration: 4 h	HF taVNS (25 Hz) significantly increased the gastric motility index compared to LF stimulation (1 Hz), primarily by enhancing the amplitude of antral peristaltic waves without affecting wave velocity or frequency, and the effect persisted for approximately 90 min post-stimulation, indicating a sustained tonic modulation of gastric motility by taVNS.
Shi et al. (2021)	IBS-C patients (*n* = 42)	Randomized controlled trial	taVNS vs. Sham-taVNS	taVNS:bilateral auricular cymba conchae Sham-taVNS: the elbow area	Frequency: 25 Hz Pulse width: 0.5 ms Intensity: 0–2 mA Duration: 4 weeks	Improved constipation, pain, psychological state, and quality of life; enhanced autonomic and rectal functions; modulated inflammation and neurotransmitters.
Liu et al. (2025)	IBS-C patients (*n* = 42)	Randomized controlled trial	taVNS vs. Sham-taVNS	taVNS:auricular cymba concha Sham-taVNS: the earlobe and antihelix	Frequency: 25 Hz Pulse width: 0.5 ms Intensity: 0–2 mA Duration: 4 weeks	improved core clinical symptoms, rectal sensation, and autonomic nervous function, and modulated cholinergic activity, gut microbiota, and related metabolite levels.
Sahn et al. (2023)	Pediatric and young adult IBD patients (CD or UC) (*n* = 22)	Randomized controlled trial	taVNS vs. Sham stimulation	taVNS:left auricular cymba conchae Sham stimulation: middle of the left calf	Frequency: 20 Hz Pulse Width: 300 μs Intensity: Titrated to tolerance Duration: 14 weeks	Effective in reducing fecal calprotectin, inducing clinical remission, and improving anxiety, with a favorable safety profile.
Huang C. H. et al. (2025)	POI patients after GI surgery (*n* = 22)	Randomized sham-controlled trial	taVNS vs. Sham	–	Duration: 15 min	Significantly shortened the time to first flatus. Enhanced the complexity and rate variability of normogastria without affecting its power ratio. Indicates that taVNS accelerates POI recovery by optimizing the complexity of gastric motility.
Ru et al. (2023)	Undergoing laparoscopic radical resection of colorectal cancer (*n* = 134)	Randomized controlled trial	tVNS vs. no stimulation	Right auricular vagal branch	Frequency: 25 Hz Intensity: 50 mA Duration: 20 min	Significantly reduced the incidence of POI. Demonstrated more regular bowel sounds at 24, 36, and 48 h postoperatively. Lower pain. Increased gastrin levels and decreased IL-6 level at 3 h after stimulation.
Huang et al. (2024)	LPRD patients (*n* = 44)	Randomized controlled trial	taVNS vs. Sham-taVNS	taVNS:bilateral auricular concha Sham-taVNS: the earlobe area	Frequency: 25 Hz Pulse width: 0.5 ms Intensity: 1.0–1.5 mA Duration: 2 weeks	Improved reflux symptoms, anxiety, and depression; increased upper and lower esophageal sphincter pressures; enhanced vagal activity.
Cai et al. (2025)	Persistent abdominal pain patients (*n* = 31)	Single-arm	taVNS	Left auricular cymba conchae	Frequency: 30 Hz Pulse width: 200 μs Duration: 20 days	Significantly reduced VAS pain scores; acute delta-band functional connectivity changes during taVNS predicted long-term pain relief, serving as a neuroplasticity biomarker.
Huang Q. et al. (2025)	Persistent abdominal pain patients (*n* = 24)	Single-arm	taVNS	Left auricular cymba conchae	Frequency: 30 Hz Pulse width: 200 μs Intensity: 0.5–2 mA Duration: 20 days	Reduced VAS and PAS scores; increased α-band power in right prefrontal regions, normalizing aberrant brain activity associated with pain.
Juel et al. (2017)	Chronic Pancreatitis patients (*n* = 20)	Randomized controlled trial	taVNS + deep slow breathing vs. Sham-taVNS	taVNS:Left auricular concha Sham-taVNS:outer earlobe	Frequency: 30 Hz Pulse Width: 250 μs Intensity: 0.1–10 mA Duration: 60 min	Increased cardiac vagal tone, but no significant effects on musculoskeletal pain sensitivity or gastroduodenal motility were observed.

FD, Functional Dyspepsia; IBS-C, Constipation-Predominant Irritable Bowel Syndrome; LPRD, Laryngopharyngeal Reflux Disease; IBD, Inflammatory Bowel Disease; CD, Crohn’s Disease; UC, Ulcerative Colitis; taVNS, Transcutaneous Auricular Vagus Nerve Stimulation; sham-ES, Sham Electrical Stimulation; tnVNS, Transcutaneous Auricular Non-Vagus Nerve Stimulation; VAS, Visual Analog Scale; PAS, Penetration-Aspiration Scale; POI, Postoperative ileus; HF, High-frequency; LF, Low-frequency.

## 5 The clinical application of taVNS in functional gastrointestinal disorders

**FIGURE 3:**
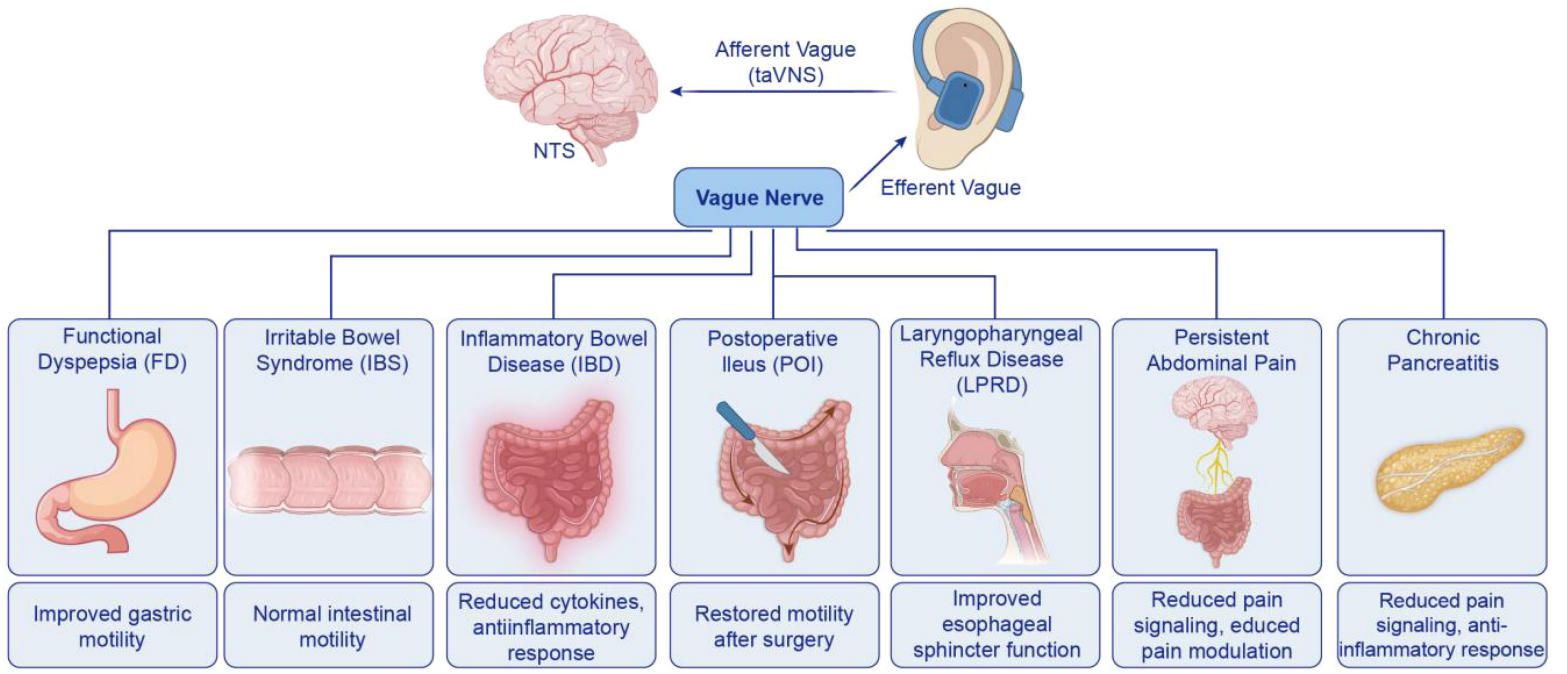
This figure summarizes the exploration of taVNS in clinical studies for various gastrointestinal disorder, including functional dyspepsia, irritable bowel syndrome, inflammatory bowel disease, postoperative intestinal obstruction, laryngopharyngeal reflux disease, and persistent abdominal pain.

In recent years, research on the application of taVNS in treating GIDs has increased significantly. Growing evidence indicates that taVNS demonstrates therapeutic effects for various gastrointestinal dysfunctions, including FD, IBS-C, persistent abdominal pain, laryngopharyngeal reflux disease (LPRD), IBD, POI, and abnormal gastrointestinal motility. Although most studies have focused on the improvement of specific gastrointestinal symptoms through taVNS, few have systematically explored its comprehensive efficacy in patients experiencing concomitant sleep disturbances, emotional issues, and reduced quality of life (Figure 3 and Table 1).

### 5.1 Therapeutic effects of taVNS on functional dyspepsia

FD is a chronic gastrointestinal disorder primarily characterized by functional disturbances of the stomach and duodenum without organic lesions. Its clinical manifestations mainly include symptoms such as early satiety, epigastric pain, and epigastric burning sensation (Ford et al., 2020). The global prevalence of this condition ranges from 11.58 to 31.50% (Black et al., 2022; Bäez et al., 2023), and it is frequently accompanied by psychological comorbidities such as depression and anxiety (Esterita et al., 2021). This not only severely compromises patients’ quality of life and work capacity but also imposes a significant economic burden on families and healthcare systems. Our analysis of key clinical trials provides robust evidence supporting the efficacy of taVNS in alleviating FD symptoms (Zhu et al., 2021; Dong et al., 2021; Shi et al., 2024; Li et al., 2025; Table 1). Multiple randomized controlled trials with sham-controlled or active comparator designs have consistently confirmed the therapeutic benefits of taVNS (Zhu et al., 2021; Dong et al., 2021; Shi et al., 2024; Li et al., 2025). Notably, Dong et al. (2021) demonstrated that taVNS sustainably improves core dyspeptic symptoms, with therapeutic effects lasting up to 12 weeks post-treatment. Additionally, one study demonstrated that taVNS significantly enhances gastric motility indices in healthy individuals. Furthermore, high-frequency stimulation was found to be more effective than low-frequency stimulation in amplifying the amplitude of gastric antral peristaltic waves (Steidel et al., 2021).

The therapeutic application of taVNS for FD appears effective across various symptom profiles and comorbidities. Studies have shown positive outcomes not only in general FD populations (Zhu et al., 2021; Dong et al., 2021; Shi et al., 2024), but also in patients with co-existing sleep disturbances (Li et al., 2025), highlighting its broad applicability. Importantly, taVNS improves both gastrointestinal-specific measures and associated psychological distress, reducing anxiety and depression scores (Zhu et al., 2021; Dong et al., 2021). Optimal stimulation parameters derived from current evidence include frequencies of 10–30 Hz (Zhu et al., 2021; Dong et al., 2021; Shi et al., 2024), pulse widths of 0.5 ms (Zhu et al., 2021; Li et al., 2025), and individualized current intensities ranging from 0.5 to 10 mA. The left auricular cymba conchae or tragus are commonly selected stimulation sites due to their rich vagal innervation (Zhu et al., 2021; Dong et al., 2021; Shi et al., 2024), though bilateral stimulation has also been employed (Li et al., 2025). Treatment duration is a key factor, with protocols of 2–4 weeks demonstrating significant improvements (Zhu et al., 2021; Dong et al., 2021; Shi et al., 2024; Li et al., 2025). Beyond symptom relief, taVNS enhances gastric accommodation (Zhu et al., 2021), normalizes gastric slow waves (Zhu et al., 2021), increases vagal activity (Zhu et al., 2021; Li et al., 2025), improves sleep quality (Li et al., 2025), supporting its multi-domain therapeutic role.

### 5.2 Therapeutic effects of taVNS on constipation-predominant irritable bowel syndrome

IBS is a common functional GID characterized by recurrent abdominal pain or discomfort associated with alterations in bowel habits or stool consistency, representing a significant condition within the spectrum of disorders of gut–brain interaction (Sebastián Domingo, 2022). Based on predominant stool patterns, IBS is clinically classified into four subtypes: diarrhea-predominant (IBS-D), IBS-C, mixed type, and unclassified (Liu et al., 2024a). Among these, IBS-C accounts for approximately one-third of all cases (Lembo et al., 2022; Hungin et al., 2003). Although not life-threatening, IBS imposes a considerable burden on patients’ quality of life, psychological wellbeing, and healthcare resource utilization (Khasawneh et al., 2024).

A single-blind randomized controlled trial (RCT) by Liu et al. (2024b) assigned 42 patients with IBS-C to either taVNS or sham stimulation for 4 weeks. Compared with sham, taVNS significantly alleviated abdominal pain and constipation severity. Mechanistically, taVNS elevated serum acetylcholine, reduced nitric oxide, enhanced vagal tone, and modulated gut microbiota and short-chain fatty acid production, indicating multi-pathway synergy in improving rectal sensation and gut-brain axis regulation. Notably, therapeutic benefits persisted post-intervention. In another RCT, Shi et al. similarly treated 42 IBS-C patients for 4 weeks (Shi et al., 2021). taVNS reduced pain scores by 64%, increased weekly spontaneous bowel movements over fourfold, and improved stool consistency. Heart rate variability analysis confirmed enhanced vagal activity. Notably, the robust clinical outcomes reported in this study led to the FDA clearance of taVNS for the treatment of IBS (Bu et al., 2026). Together, these studies demonstrate that non-invasive taVNS concurrently relieves abdominal pain and constipation while physiologically modulating intestinal sensorimotor function and autonomic balance.

### 5.3 Therapeutic effects of taVNS on inflammatory bowel disease

IBD, encompassing Crohn’s disease (CD) and ulcerative colitis (UC), is a chronic and relapsing inflammatory condition that affects a growing number of pediatric and young adult patients worldwide, leading to significant morbidity and healthcare burden (Bruner et al., 2023; Flynn and Eisenstein, 2019). Current pharmacologic treatments, though effective for many, often entail toxicity risks, high costs, and incomplete mucosal healing (Sairenji et al., 2017; Gaidos and Hashash, 2025). In recent years, taVNS has emerged as a novel non-invasive neuromodulation approach with promising anti-inflammatory potential. Sahn et al. (2023) conducted a proof-of-concept trial involving 22 pediatric and young adult patients with mild-to-moderate IBD (Sahn et al., 2023). Participants were initially randomized to receive active taVNS or sham stimulation in a crossover phase, followed by an open-label extension with twice-daily taVNS for up to 16 weeks. taVNS significantly reduced fecal calprotectin levels in 64.7% of patients, with UC patients showing a median reduction of 81%. By week 16, half of CD patients and one-third of UC patients achieved clinical remission. taVNS was well tolerated with no serious adverse events, and anxiety scores also improved. These findings support taVNS as a safe, effective non-pharmacological intervention capable of modulating inflammation and improving clinical outcomes in pediatric IBD, suggesting its potential as an adjunct or alternative to conventional therapies.

### 5.4 Therapeutic effects of taVNS on postoperative ileus

POI is a common complication following abdominal surgery, which significantly prolongs hospital stay and increases the healthcare burden (Ru et al., 2023; Huang C. H. et al., 2025). A recent randomized controlled trial demonstrated that a single preoperative session of low-intensity taVNS significantly reduces POI incidence in patients undergoing laparoscopic colorectal cancer resection (Ru et al., 2023). The intervention group received 20 min of taVNS at the right tragus before anesthesia, resulting in a markedly lower POI rate versus controls. These patients exhibited earlier and more regular return of bowel sounds, lower postoperative pain scores, increased serum gastrin, and reduced IL-6 levels at 3 h post-stimulation. The authors propose that taVNS restores gastrointestinal motility via nucleus tractus solitarius activation, rebalancing autonomic tone and dampening systemic inflammation. Similarly, Huang et al. found that daily 15-min taVNS accelerated time to first flatus in gastrointestinal surgery patients. Electrogastrography indicated that taVNS enhanced the complexity and variability of gastric slow-wave activity without increasing its overall power, suggesting modulation of gastric dynamics through gut–brain axis pathways rather than mere amplification of electrical signals (Huang C. H. et al., 2025). These findings support taVNS as a promising preventive strategy for POI, though larger trials are needed to confirm its efficacy.

### 5.5 Therapeutic effects of taVNS on laryngopharyngeal reflux disease

LPRD is a clinical syndrome where gastric or duodenal contents reflux above the upper esophageal sphincter, causing symptoms and signs in the larynx and adjacent airways (Mishra et al., 2020). Common symptoms include globus sensation, chronic throat clearing, hoarseness, throat pain, and persistent cough. Laryngoscopic findings may show supraglottic mucosal thickening, a shallow laryngeal vestibule, or contact granulomas (Campagnolo et al., 2014). A recent randomized sham-controlled pilot trial found that twice-daily taVNS for 2 weeks effectively alleviated both pharyngeal symptoms and psychological distress in LPRD patients (Huang et al., 2024). Compared to sham stimulation, active taVNS applied bilaterally to the auricular concha for 30 min per session led to significantly greater reductions in Reflux Symptom Index scores and decreased anxiety and depression scores. Treatment also enhanced upper and lower esophageal sphincter resting pressures and increased heart rate variability, reflecting improved parasympathetic tone. These improvements are likely mediated through vagally reinforced esophageal barrier function and central modulation of autonomic balance, supporting taVNS as a promising intervention for LPRD.

### 5.6 Therapeutic effects of taVNS on persistent abdominal pain

Persistent abdominal pain (PAP) is characterized by chronic discomfort between the chest and pelvis, often severely impairing quality of life and contributing to anxiety and depression. Prolonged pain can heighten visceral hypersensitivity and central sensitization, increasing the risk of comorbid chronic pain conditions (Simoni et al., 2020). Management of PAP remains clinically challenging, as existing pharmacotherapies often lack specificity or comprehensive mechanism coverage, leading to limited efficacy or notable adverse effects in many patients (Coffin and Duboc, 2022). In a randomized sham-controlled pilot trial, Huang et al. treated 24 persistent abdominal pain (PAP) patients with twice-daily 30-min taVNS for 20 days (Huang Q. et al., 2025). Active taVNS significantly reduced both Visual Analog Scale and Pain Anxiety Scale scores, indicating concurrent pain and emotional relief. Neurophysiologically, taVNS enhanced α-band power in the right lateral and dorsomedial prefrontal cortices—regions negatively correlated with pain intensity. This α-band modulation persisted post-treatment, suggesting sustained cortical reorganization. Similarly, Cai et al. (2025) combined acute taVNS-EEG with a 20-day treatment regimen in 31 PAP patients. Pain scores were significantly reduced after intervention. Acute taVNS broadly modulated delta-band connectivity, notably decoupling the salience/attention network from somatomotor regions. Using connectome-based predictive modeling, acute changes in the weighted phase-lag index during taVNS accounted for approximately 25.5% of the variance in long-term pain relief, indicating that early neurophysiological responses can predict treatment outcomes and supporting taVNS-induced neuroplasticity within pain networks.

### 5.7 Therapeutic effects of taVNS on chronic pancreatitis

Chronic pancreatitis (CP) is a fibro-inflammatory disorder often accompanied by persistent pain, which markedly reduces quality of life and may contribute to anxiety and depression (Beyer et al., 2020). Current pain management in CP remains largely pharmacologically based (Yang and Forsmark, 2017). However, analgesic monotherapy frequently yields suboptimal relief, prompting the use of opioid and gabapentinoid combinations. These regimens are limited by adverse effects, including opioid-induced gastrointestinal dysmotility, and a trade-off between analgesia and tolerability (O’Brien and Omer, 2019). Juel et al. (2017) conducted a randomized single-blind crossover trial in 20 chronic pancreatitis patients, comparing a single 60-min session of taVNS combined with deep slow breathing against sham stimulation (Juel et al., 2017). While the intervention significantly increased cardiac vagal tone, it did not improve pressure pain thresholds or gastroduodenal motility. Furthermore, conditioned pain modulation was weakened in the taVNS group, indicating impaired descending pain inhibition.

### 5.8 Therapeutic prospects of taVNS in diarrhea-related gastrointestinal disorders

The current clinical evidence for taVNS in GIDs is heavily skewed toward conditions marked by hypomotility, blunted vagal tone, or chronic mucosal inflammation, such as FD, IBS-C, IBD, POI, and persistent abdominal pain. Diarrhea-predominant disorders have received markedly less attention, and to date no dedicated RCT of taVNS has been published in IBS-D, functional diarrhea, bile-acid diarrhea, microscopic colitis, or chemotherapy- or radiation-induced diarrhea. This is an important gap that warrants explicit discussion. Several lines of evidence suggest that taVNS is likely to be indicated in these populations. The FDA-cleared PENFS device, which engages the same auricular vagal target, reduced abdominal pain without exacerbating diarrhea in adolescents with abdominal-pain-related DGBIs, including IBS-D and mixed IBS (Kovacic et al., 2017; Krasaelap et al., 2020). Mechanistically, taVNS is a selective prokinetic: in healthy volunteers it normalizes gastric dysrhythmias toward physiological patterns rather than uniformly accelerating transit (Steidel et al., 2021). The non-canonical CAP engaged by taVNS would also be expected to attenuate, not aggravate, inflammation-driven diarrhea (Bonaz et al., 2017; Sahn et al., 2023). Caution remains warranted, however, as vagal-cholinergic activation can accelerate transit and intestinal secretion; one IBS-C trial reported a single case of intolerable diarrhea on day 1 of active taVNS that resolved with continued treatment (Liu et al., 2024b). Future research should prioritize: (1) sham-controlled RCTs in IBS-D and functional diarrhea using lower-frequency, lower-intensity, or shorter-duration protocols; (2) pilot trials in inflammation-driven diarrhea such as microscopic colitis and checkpoint-inhibitor or chemotherapy-induced colitis; and (3) prespecified stool-form and bowel-frequency safety endpoints with predefined stopping rules, alongside responder-stratification by baseline vagal tone and inflammatory biomarkers (section 7.4).

## 6 The underlying mechanism of taVNS in gastrointestinal disorders

**FIGURE 4:**
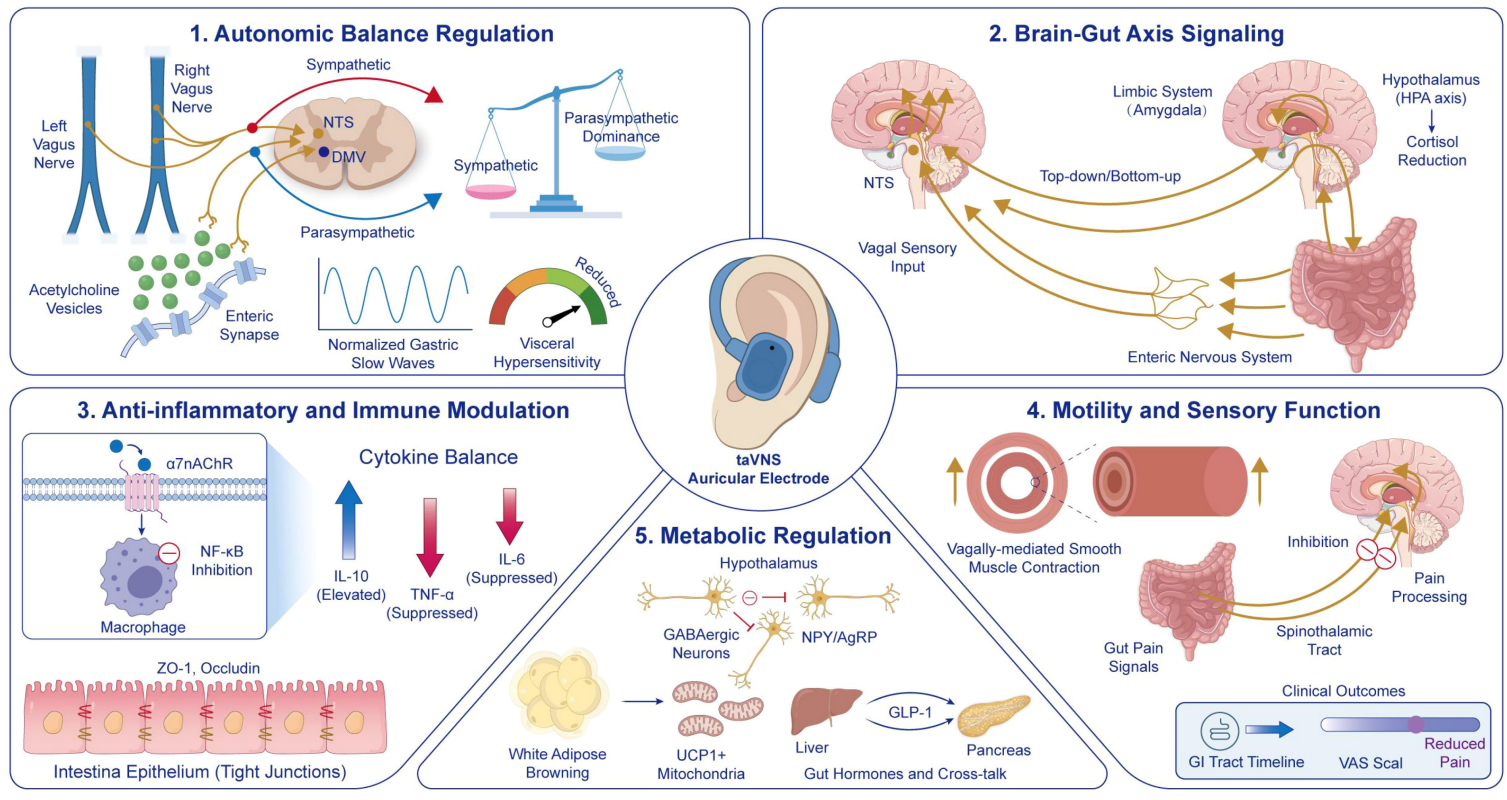
Illustrates the comprehensive mechanism of taVNS in treating gastrointestinal disorders: auricular stimulation activates the vagus nerve, with signals integrated via the NTS and translated into therapeutic effects through five synergistic pathways—regulating vagal activity to improve autonomic balance, modulating the brain–gut axis to influence central and peripheral signaling, exerting anti-inflammatory and immunomodulatory effects, enhancing gastrointestinal motility and sensory function, and regulating energy metabolism along with obesity-related gastrointestinal dysfunction.

The therapeutic effects of taVNS in GIDs are mediated by multiple interrelated mechanisms, with the core mechanism being the targeted regulation of the vagus nerve-brain-gut axis system. Its primary mechanisms of action can be summarized into the following six interconnected aspects (Figure 4 and Table 2): enhancing vagal tone to restore autonomic balance; regulating central-peripheral signaling; exerting anti-inflammatory and immunomodulatory effects; improving gastrointestinal motility and visceral sensory function; regulating energy metabolism and obesity-related gastrointestinal dysfunctions. These mechanisms synergistically intervene in key pathophysiological pathways to collectively promote gastrointestinal functional recovery. The following sections will elaborate on the specific action mechanisms of each component, supported by evidence from animal model studies.

**TABLE 2:** The underlying mechanism of taVNS in gastrointestinal disorder.

References	Rodent models	Stimulation site	taVNS parameter	Key biomarkers/ effects	Results and conclusion
Hou et al. (2020)	Rat FD model	Bilateral auricular concha cavity	Duration: 7 days	MTL, CCK, GLP-1; IL-4, IL-10, IL-1β; gastric sensitivity	Reduced gastric sensitivity, normalized brain-gut peptide levels, and exerted anti-inflammatory effects, suggesting a therapeutic role in FD via vagal modulation.
Hou et al. (2021)	Rat FD model	Unilateral auricular concha	Intensity: 0.5 mA Duration: 14 days	HRV; Ach; M3R; EMG; AWR score	Increased vagal activity, improved autonomic balance, elevated Ach and M3R expression, and reduced gastric sensitivity, suggesting a mechanism involving vagal modulation for treating FD.
Hou et al. (2022)	Rat FD model	Unilateral cymba concha and cavum concha	Frequency:20/100 Hz Intensity:2 mA Duration: 14 days	CRF, CRHR1, ACTH, Corticosterone; Gastric emptying, OFT behavior	Improved gastric motility and depression-like behavior, likely through inhibition of the HPA axis and CRF pathway, providing evidence for taVNS as a non-invasive therapy for FD.
Hou et al. (2021)	Rat FD model	Unilateral cymba concha and cavum concha	Frequency: 20/100 Hz Intensity: 2 mA Duration: 14 days	Tight junction proteins: ZO-1, Occludin general condition score, 3 h food intake, gastric emptying rate	Improved general condition, food intake, and gastric motility in FD rats. It up-regulated the expression of tight junction proteins ZO-1 and Occludin in the duodenum, suggesting a therapeutic role by repairing the intestinal mucosal barrier.
Hou et al. (2023)	Rat FD model	Cymba conchae and cavum conchae	Frequency: 100 Hz Intensity: 0.5 mA Duration: 14 days	TNF-α, IL-6, IL-1β, NF-κBp65; CRF, CRF1, ACTH, corticosterone; HRV, Gastric ACh, M3R; DSG2, Occludin; EMG, Gastric emptying, OFT	Improved visceral hypersensitivity, gastric motility, and depression-like behaviors. Mechanisms involved anti-inflammatory effects via the cholinergic pathway, inhibition of the HPA axis, and enhancement of duodenal mucosal integrity. These integrative effects were mediated by the vago-vagal pathway, as evidenced by vagotomy abolishing the benefits.
Zhou et al. (2024)	Rat FD model	Bilateral cymba conchae and cavum conchae	Frequency: 2 Hz Intensity: 0.7–1.0 mA Duration: 5 days	Gastric NGF; NGF, TrkA, PLC-γ, TRPV1; p-TrkA, p-PLC-γ	Alleviated gastric hypersensitivity, reduced peripheral gastric NGF, and downregulated the central NGF/TrkA/PLC-γ signaling pathway in the NTS, suggesting a molecular mechanism for its therapeutic effect via the brain-gut axis.
Han et al. (2022)	Rat FD model	Bilateral auricular	Frequency: 100 Hz Intensity: 0.5 mA Duration: 14 days	ACh, α7nAChR, IL-6, IL-1β, TNF-α, NF-κB p65; Gastric sensitivity, Gastric emptying rate	Reduced gastric sensitivity, promoted gastric emptying, upregulated ACh and α7nAChR, and suppressed NF-κB pathway and pro-inflammatory cytokines, suggesting a mechanism involving the cholinergic anti-inflammatory pathway.
Zhang et al. (2021b)	Rat OIC model	Right auricular concha	Frequency: 25 Hz Pulse width: 0.5 ms Intensity: 1–2 mA Duration: 7 days	Fecal pellets, water content, colon transit, c-fos, HRV, ChAT, nNOS, GDNF, c-kit	Improved constipation, accelerated colon transit, increased vagal efferent activity, activated NTS/DMV, and restored enteric neural and ICC functions, indicating a central-vagal efferent pathway mechanism for treating OIC.
Liu et al. (2024a)	Mouse IBS-C model	Left auricular concha	Frequency: 25 Hz Pulse width: 0.6 ms Duration: 14 days	Fecal pellets, water content, GI transit, AWR score, gut microbiota, c-kit	Improved constipation symptoms, accelerated GI transit, reduced visceral hypersensitivity, restored beneficial gut microbiota, and increased ICC networks, suggesting therapeutic potential for IBS-C via brain-gut-microbiome axis.
Hesampour et al. (2024)	Mouse DSS/DNBS colitis models	Bilateral cymba concha of ears	Frequency: 20 Hz Intensity: 1 mA Pulse width: 500 μs Duration: 4–6 days	IL-1β, TNF-α, TGF-β, IL-10, Mip1β, MMP9, MMP2, Nos2, Bax, Bak1, Casp8, Bad	Improved disease activity, reduced pro-inflammatory and pro-apoptotic markers, and enhanced anti-inflammatory and pro-survival markers in DSS-induced colitis, but effects were model-dependent, showing limited efficacy in DNBS-induced colitis.
Hong et al. (2019)	Mouse POI and endotoxemia models	Right auricular concha	Frequency: 10 Hz Intensity: 1 mA Duration: 10 min	c-fos in NTS/DMV; IL-6, MCP-1, IL-1β, TNF-α; MPO^+^ leukocytes; GI transit	Activated NTS and DMV, reduced intestinal and systemic inflammation, improved GI transit, and prevented POI and endotoxemia; effects were vagus nerve-dependent.
Zhang Y. et al. (2024)	ZDF diabetic rats	Bilateral auricular concha	Frequency: 2/15 Hz Intensity: 2 mA Duration: 3 weeks	Fasting blood glucose, plasma melatonin, intestinal melatonin receptors	Reduced blood glucose and body weight, upregulated plasma melatonin and intestinal melatonin receptor expression, indicating a hypoglycemic effect mediated by gut melatonin system.
Zhang et al. (2021b)	SD rats with loperamide-induced GI dysmotility	Right auricular concha	Frequency: 5 Hz Intensity: 1–2 mA Duration: 7 days	PP, NE; Gastric emptyin, small intestinal transit, GI slow wave, c-kit	Improved GE and SIT, normalized slow waves, increased PP and decreased NE, restored ICC density, suggesting taVNS ameliorates opioid-induced dysmotility via vagal efferent and ICC pathways.
Xin et al. (2025)	HFD-induced obese SD rats	Bilateral auricular concha	Frequency: 2/5 Hz Intensity: 1 mA Duration: 6 weeks	UCP1, SLC25A44, PPM1K, BCKDK; BAT thermogenesis, mitochondrial morphology, WAT/BAT mass	Ameliorated obesity by enhancing BCAA metabolism via the UCP1/SLC25A44 pathway, promoting adipose tissue browning and thermogenesis.
Yu et al. (2021)	ZDF rats	Bilateral auricular concha	Frequency: 15 Hz Intensity: 2 mA Duration: 4 weeks	Hypothalamic P2Y1R expression; Body weight; Food intake	Attenuated weight gain without reducing food intake, likely by increasing energy expenditure via downregulation of hypothalamic P2Y1R expression in astrocytes.
Zhang et al. (2025)	HFD-induced obese Balb/c mice	Bilateral cymba conchae	Frequency: 10 Hz Duration: 7 days	Orexin A; food intake, body weight, fat pad weight, blood lipids	Activated the PVN-GABA-LH circuit to suppress orexin, thereby reducing food intake and body weight, indicating a central appetite-control mechanism.
Yu et al. (2022)	Zucker diabetic fatty rats	Bilateral auricular concha	Frequency: 15 Hz Intensity: 2 mA Pulse width: 0.5 ms Duration: 4 weeks	P2X7R expression in limbic regions; blood glucose; depressive-like behaviors	Reduced blood glucose, attenuated weight gain, reversed depressive-like behaviors, and suppressed limbic P2X7R expression, primarily in astrocytes, suggesting a potential mechanism for its antidepressant effect in diabetic depression.

FD, Functional Dyspepsia; OIC, opioid-induced constipation; IBS-C, Constipation-Predominant Irritable Bowel Syndrome; DSS, dextran sulfate sodium; DNBS, dinitrobenzene sulfonic acid; POI, ostoperative ileus; ZDF, Zucker diabetes fatty; SD, Sprague-Dawley; GI, Gastrointestinal dysmotility; HFD, high-fat diet; HRV, Heart Rate Variability; ICC, interstitial cells of Cajal.

### 6.1 Regulation of vagus nerve activity to improve autonomic nervous system balance

By stimulating the concha region, taVNS activates vagal pathways, enhancing cardiac vagal activity and ameliorating sympathetic-vagal imbalance (Wang W. et al., 2025). Its autonomic regulation operates primarily through two interconnected pathways. First, the central vagus-vagus reflex constitutes the core mechanism: auricular stimulation sends afferent signals to the brainstem’s NTS and dorsal motor nucleus of the vagus (DMV), boosting vagal efferent outflow (Hong et al., 2019). This reflex restores autonomic equilibrium by elevating parasympathetic tone, increasing high-frequency (HF) power in heart rate variability, and lowering the low-frequency (LF)/HF ratio (Hou et al., 2021). Second, taVNS modulates gastrointestinal autonomic function via the enteric nervous system. Enhanced vagal efferent activity upregulates choline acetyltransferase (ChAT) and acetylcholine release, improving motility rhythms and reducing visceral hypersensitivity through balanced excitatory-inhibitory neuronal coordination (Zhang et al., 2021a; Du et al., 2024).

In pathological conditions like FD and OIC, autonomic imbalance—marked by reduced vagal and heightened sympathetic tone—is common. taVNS counteracts this by significantly improving heart rate variability profiles in FD models, concurrently raising gastric acetylcholine and M3 receptor levels, which correlates with attenuated gastric sensitivity and improved function (Hou et al., 2021). Similarly, in constipation models, taVNS restores vagal efferent activity, increases HF power, and accelerates colonic transit—effects abolished by vagotomy or cholinergic blockade, confirming vagal-cholinergic pathway dependence (Zhang et al., 2021b). Moreover, taVNS promotes intestinal neural plasticity via glial-derived neurotrophic factor (GDNF) upregulation, supporting long-term autonomic stability. Activation of the NTS/DMV pathway further mediates taVNS’s anti-inflammatory and prokinetic effects, underscoring its vagus-dependent mechanism (Hong et al., 2019). Thus, taVNS corrects autonomic imbalance through synergistic central and enteric cholinergic regulation, offering a promising non-invasive strategy for functional GIDs.

### 6.2 Regulation of the brain-gut axis to influence central and peripheral signaling

The brain–gut axis functions as a critical bidirectional communication network, integrating neural, endocrine, and immune signals between the central nervous system and the gastrointestinal tract. It regulates not only gastrointestinal perception, motility, and local immunity but also emotional, stress, and cognitive functions (Hou et al., 2022). In GIDs, dysregulated motility and visceral hypersensitivity often coincide with emotional disturbances, excessive stress responses, and cognitive changes. These components interact, creating a vicious cycle that exacerbates symptoms (Hou et al., 2023; Zhou et al., 2024), making interventions targeting the brain–gut axis essential for treatment. As a non-invasive neuromodulation technique, taVNS stimulates auricular vagal afferents, relaying signals to the NTS in the brainstem—a key hub for visceral sensory integration (Yue et al., 2023). NTS activation modulates autonomic centers and projects to higher brain regions, including the hypothalamus, amygdala, and prefrontal cortex, thereby broadly influencing emotional, stress, and gastrointestinal autonomic responses (Liu et al., 2020). Importantly, taVNS downregulates overactive nerve growth factor signaling within the NTS, providing a molecular basis for its ability to alleviate gastric hypersensitivity through central analgesic pathways (Zhou et al., 2024).

Beyond the brainstem, taVNS exerts significant effects on higher regulatory centers. For example, in obese models, taVNS activates GABAergic neurons in the hypothalamic paraventricular nucleus, enhancing inhibitory projections to feeding centers and reducing appetite peptide release, which supports weight control (Zhang et al., 2025). This illustrates taVNS’s capacity to modulate energy-regulating neural circuits. Moreover, taVNS achieves integrated “brain–gut co-regulation” by effectively suppressing the hypothalamic–pituitary–adrenal axis (Li et al., 2020). In stress-induced functional dyspepsia models, it reduces hypothalamic CRF and peripheral corticosterone, alleviating depressive-like behaviors and improving gastric motility (Hou et al., 2022; Hou et al., 2023). By breaking the stress–gut–mood cycle, taVNS offers a neuroendocrine rationale for treating GIDs with emotional comorbidities. Thus, through coordinated modulation of the brainstem NTS, hypothalamic feeding/stress centers, and associated molecular pathways, taVNS achieves holistic brain–gut axis regulation, highlighting its broad therapeutic promise in disorders of gut–brain interaction.

### 6.3 Anti-inflammatory and immunomodulatory effects

GIDs progression is closely linked to dysregulated inflammatory mediators. While factors like TNF-α, IL-1β, and IL-6 normally support immune defense and tissue repair, their dysregulation can drive persistent inflammation, mucosal barrier disruption, edema, and apoptosis, thereby advancing conditions such as gastritis and IBD (Bassotti et al., 2014; Bjarnason et al., 2018; Hijos-Mallada et al., 2022).

taVNS exerts notable anti-inflammatory and barrier-protective effects in preclinical models. Mechanistically, it rebalances inflammatory signaling by downregulating pro-inflammatory cytokines and upregulating anti-inflammatory mediators. Central to this is the non-canonical CAP: taVNS enhances acetylcholine release, which activates α7 nicotinic acetylcholine receptors to suppress NF-κB signaling and limit pro-inflammatory cytokine production (Han et al., 2022; Hou et al., 2023). Studies in FD and colitis models consistently demonstrate reduced levels of TNF-α, IL-6, IL-1β, and NF-κB p65 following taVNS (Hou et al., 2020; Hou et al., 2023; Hesampour et al., 2024). Notably, efficacy may vary by model, as taVNS showed robust effects in DSS-induced colitis but limited impact in DNBS-induced colitis, suggesting context-dependent activity (Hesampour et al., 2024). Beyond cytokine modulation, taVNS strengthens intestinal barrier integrity by upregulating tight-junction proteins ZO-1 and Occludin, thereby reducing mucosal permeability (Hou et al., 2021). This dual action—simultaneously dampening inflammation and reinforcing epithelial structure—provides a consolidated pathophysiological rationale for taVNS in treating functional GIDs.

### 6.4 Improvement in gastrointestinal motility and sensory function

Delayed gastric emptying and slow intestinal transit are core motility-related pathological features of GIDs (Bassotti et al., 2014; Di Rosa et al., 2023). Reduced motility stems from three key defects: abnormal smooth muscle contractility, disrupted enteric nervous system (ENS) regulation, and impaired interstitial cell of networks–manifesting clinically as postprandial fullness, bloating, and defecation difficulty (Kaji and Hori, 2023; Akahoshi et al., 2018). Restoring normal transit is thus critical to easing patient burden.

taVNS enhances gastric/intestinal smooth muscle contractility and accelerates transit via vagus-dependent pathways, directly relieving motility-related symptoms. In FD models, Hou et al. (2020) demonstrated taVNS reduces gastric sensitivity and promotes emptying, improving postprandial fullness/early satiety (Hou et al., 2020; Hou et al., 2022). For OIC, Zhang et al. found taVNS accelerated colonic transit, activated the ENS, upregulated enteric acetylcholinesterase/glial cell-derived neurotrophic factor (GDNF), and restored interstitial cells of Cajal (ICC) function—alleviating constipation (Zhang et al., 2021b). In IBS-C mice, Liu et al. (2024a) validated taVNS boosts motility, speeds fecal excretion, rebuilds ICC networks, and eases visceral hypersensitivity (Liu et al., 2024a). Further, in loperamide-induced motility disorders, Zhang et al. showed taVNS normalized slow-wave activity, increased ICC density in gastric antrum/colon, and reversed drug-induced abnormalities, confirming its regulatory role in motility (Zhang et al., 2021a).

Visceral hypersensitivityd drugnsity in gastric antrum/colon, and reversed drugeral hypersensitivity (persensylcholto normal/mild gastrointestinal stimuli (Mertz, 2003) and linked to overactive central pain pathways and dysregulated inflammatory mediators (Bjarnason et al., 2018). taVNS relieves this by suppressing excessive neural responses to visceral stimuli and reducing hyperalgesia. For example: in FD models, it attenuated gastric distension-induced hypersensitivity via the non-canonical CAP and reduced pro-inflammatory factor release (Hou et al., 2023); in IBS-C models, it eased colorectal distension-induced pain behaviors (Liu et al., 2024a). These data show taVNS modulates visceral sensory pathways at multiple levels, offering a safe, non-pharmacological option for pain relief in functional GIDs.

### 6.5 Regulation of energy metabolism and obesity-related gastrointestinal dysfunction

Obesity is often accompanied by disturbances in energy metabolic homeostasis and related GIDs, manifested as abnormal feeding behavior, gastrointestinal hormone imbalance, and disruption of the dynamic equilibrium between energy storage and expenditure (Park et al., 2022; Cypess et al., 2025). This abnormal metabolic state not only exacerbates insulin resistance, dyslipidemia, and visceral fat accumulation but is also closely associated with the onset and progression of various functional GIDs (Cypess et al., 2025; Lynch and Adams, 2014; Valassi et al., 2008). Therefore, restoring energy metabolism balance and improving gastrointestinal function are central strategies in managing obesity and its complications.

Recent studies indicate that taVNS plays a significant role in regulating energy metabolism and feeding behavior. In a high-fat diet-induced obese rat model, Xin et al. (2025) demonstrated that taVNS promotes the “browning” of white adipose tissue by upregulating the expression of uncoupling protein 1 and the mitochondrial transporter SLC25A44 (Xin et al., 2025). This enhances mitochondrial structural and functional integrity, thereby increasing energy expenditure, reducing body weight, and improving glucose and lipid metabolism disorders. At the central regulatory level, taVNS acts on key brain regions governing energy and feeding control, such as the hypothalamus. Yu et al. (2021) found that in Zucker diabetic fatty rats, taVNS effectively suppressed weight gain by downregulating hypothalamic purinergic receptor P2Y1R expression without affecting food intake, suggesting its weight-loss effect may be related to enhanced energy expenditure mechanisms (Yu et al., 2021). Furthermore, Zhang et al. (2025) revealed in a high-fat diet mouse model that taVNS suppresses appetite by activating gamma-aminobutyric acid projections from the paraventricular nucleus to the lateral hypothalamus, thereby inhibiting the secretion of the appetite-stimulating neuropeptide orexin (Zhang et al., 2025). This study elucidates the central mechanism of taVNS in appetite regulation at the neural circuit level.

Moreover, taVNS demonstrates synergistic benefits in regulating metabolic and emotional comorbidities. Zhang Y. et al. (2024) reported that in Zucker diabetic rats, taVNS improves hyperglycemia by upregulating intestinal melatonin receptor expression and promoting melatonin secretion (Zhang Y. et al., 2024). Concurrently, Yu et al. (2022) observed that taVNS not only lowers blood glucose and reduces body weight but also reverses depression-like behaviors while suppressing expression of the inflammatory receptor P2X7R in limbic brain regions (Yu et al., 2022). This suggests that taVNS exerts multi-target synergistic effects in regulating glucose metabolism and intervening in mood disorders. In summary, taVNS demonstrates multidimensional, integrative therapeutic potential in ameliorating obesity-related energy metabolism disorders and GIDs through promoting peripheral adipose tissue thermogenesis, regulating central appetite circuits, and holistically modulating the neuroendocrine-immune network. This offers novel insights for non-pharmacological interventions in obesity and its complications.

## 7 Limitations of the current study and future prospects

### 7.1 The lack of standardized stimulation parameters hinders clinical translation and comparative studies

Currently, standardization of taVNS in clinical applications for digestive system disorders remains significantly challenging, with considerable heterogeneity in stimulation parameters, stimulus duration, and treatment course settings, and treatment course settings. As shown in Table 1, parameters used in studies targeting FD or IBS-C vary considerably: frequencies range from 10 to 30 Hz, intensities span 0.5–10 mA, stimulation sites primarily focus on the concha or tragus regions, and treatment durations extend from 2 weeks to over 4 weeks. This “parameter jungle dilemma” not only complicates cross-study comparisons and meta-analyses but also undermines the reproducibility of clinical outcomes. For instance, in healthy subjects, high-frequency versus low-frequency transnasal vagal stimulation produced markedly different effects on gastric motility (Steidel et al., 2021), indicating that parameter selection critically influences physiological responses and therapeutic efficacy.

This lack of standardization primarily stems from insufficient understanding of the dose-response relationship of vagus nerve stimulation. Effective treatment relies on fully activating auricular vagus nerve fibers to modulate brainstem nuclei such as the solitary tract nucleus. However, quantitative models linking stimulation parameters to neurophysiological or clinical outcomes remain in their infancy. Future translational research should adopt a multi-tiered strategy: at the basic science level, integrating computational modeling with neuroimaging techniques to map neural activation and central responses across varying parameters (Kreisberg et al., 2021); in preclinical studies, systematically evaluating parameter-dependent pathways within disease models; and in clinical trials, employing adaptive or model-guided designs to determine optimal parameter combinations for specific diseases and patient subgroups. Ultimately, establishing a standardized, tiered, disease-specific parameter framework through interdisciplinary collaboration will propel taVNS from an empirical tool toward precision medicine.

### 7.2 The clinical study design has limitations, and the level of evidence requires improvement

Clinical trials of taVNS for GIDs still face challenges such as limited sample sizes, short follow-up periods, and significant design heterogeneity. These issues manifest as inconsistent sham stimulation controls and diverse outcome measures. Most current studies are single-center, resulting in insufficient statistical power that limits the generalizability of findings and hinders thorough assessment of long-term efficacy and safety. Future studies should prioritize large-sample, multicenter, randomized double-blind designs, establish standardized sham-stimulation controls, and incorporate adaptive trial designs to optimize parameter selection. Endpoint assessment should integrate patient-reported outcomes with objective markers such as gastrograms, heart rate variability, and inflammatory biomarkers, supplemented by long-term follow-up to comprehensively evaluate sustained efficacy. Furthermore, study protocols must be pre-registered with high transparency, detailing stimulation parameters and blinding implementation. Finally, patient stratification studies are essential to develop predictive models based on vagal function, disease subtypes, or biomarkers, thereby advancing taVNS from a universal treatment to a personalized precision intervention paradigm.

### 7.3 Mechanism and clinical research face a “translation gap”

Despite preliminary insights from animal studies on the multi-pathway mechanisms through which taVNS modulates GIDs–including enhancing vagal tone, regulating brain–gut axis signaling, exerting anti-inflammatory effects, and improving motility and sensory function–a notable “translational gap” remains between preclinical findings and clinical application. Animal models differ from human disease in etiology, progression, and systemic complexity, and many observed effects lack robust validation in clinical studies. For instance, while taVNS clearly inhibits NF-κB signaling and upregulates tight-junction proteins in animals via the non-canonical CAP, its anti-inflammatory and barrier-repairing actions in patients still await large-scale, multicenter evidence (Hesampour et al., 2024).

Current clinical trials primarily focus on symptom improvement as the endpoint, with insufficient monitoring of mechanism-related biomarkers. This makes it difficult to establish direct links with molecular pathway changes observed in animal studies. Simultaneously, variations in stimulation parameters pose challenges for systematically integrating mechanism exploration with efficacy assessment. Future research should establish more comprehensive translational pathways by integrating mechanism validation into clinical trial design. By combining multi-omics analysis, neuroimaging, and dynamic physiological monitoring, we may bridge the evidence gap between animal mechanisms and clinical efficacy, driving a substantive leap from basic research to clinical practice.

### 7.4 Significant individual variability in treatment outcomes and lack of precision medicine protocols

The clinical efficacy of taVNS in treating GIDs exhibits significant interindividual variability due to anatomical differences in vagal innervation, baseline autonomic tone, and disease heterogeneity. Current standardized protocols therefore often fail to optimize outcomes, highlighting the need for precision taVNS approaches. Future strategies should integrate pre-treatment biomarkers—such as heart rate variability, EEG patterns, genetic profiles, or microbiome signatures—to predict responsiveness. During therapy, closed-loop systems could dynamically adjust stimulation parameters, such as frequency and intensity, based on real-time physiological feedback, including measures of gastric activity, cortisol levels, and inflammatory markers. Subtype-specific protocols should also be developed—for example, low-frequency stimulation for visceral hypersensitivity and high-frequency stimulation for motility disorders. Implementing a multidimensional, closed-loop precision framework—encompassing assessment, prediction, intervention, and optimization—is essential to advance taVNS from a uniform intervention toward personalized, reliable therapeutics.

### 7.5 Equipment and home treatment protocols remain inconsistent, limiting accessibility and adherence

The clinical translation and home application of taVNS are impeded by the lack of standardization in both device design and treatment protocols. Commercially available devices vary in electrode form, materials, and fit, which affects stimulation consistency and targeting accuracy. Output parameters also differ across manufacturers and even between modes on the same device, hampering the reproducibility of clinical results. Portable, user-friendly devices for home use remain underdeveloped, with their safety and efficacy yet to be validated, thus limiting accessibility outside clinical settings. Treatment protocols are equally heterogeneous, with no consensus on stimulation site, duration, frequency, or parameter selection. The absence of simplified, personalized guidance for home use reduces adherence and complicates long-term management. To overcome these barriers, multidisciplinary efforts are needed to establish device certification standards and develop integrated biofeedback-enabled systems for personalized dosing. Standardized, evidence-based treatment guidelines should be formulated alongside patient education and remote monitoring tools, ultimately advancing taVNS into a feasible, reliable, and accessible home-based therapy.

### 7.6 Applicability and safety considerations across gastrointestinal disorder subtypes

A practical question for clinical translation is whether taVNS is appropriate for GIDs in which excessive vagal tone or accelerated transit might theoretically aggravate symptoms—most notably gastritis, gastroesophageal reflux, and IBS-D. Although classical physiological studies in animals demonstrated that direct cervical vagal stimulation augments basal gastric acid output and potentiates distension- or peptone-induced acid secretion via cholinergic activation of parietal cells (Naseri et al., 1991; Noto et al., 1997), the situation with taVNS appears substantially different. taVNS preferentially recruits afferent fibers of the auricular branch of the vagus nerve and engages a vago-vagal reflex through the NTS and DMV, rather than producing the maximal, non-physiological efferent volley elicited by direct cervical stimulation (Badran et al., 2019; Hong et al., 2019). To date, no clinical trial of taVNS in functional dyspepsia, IBS, IBD, LPRD, postoperative ileus, or persistent abdominal pain has reported clinically meaningful increases in gastric acid secretion, new-onset acid-related dyspepsia, or worsening of pre-existing reflux. On the contrary, in a randomized controlled trial of LPRD—a condition in which acid reflux is the principal driver of symptoms—active taVNS reduced reflux symptom indices and increased upper and lower esophageal sphincter pressures rather than aggravating reflux (Huang et al., 2024), suggesting that any pro-secretory action is outweighed by improved barrier function and esophageal motility. Moreover, the non-canonical CAP activated by taVNS would, if anything, be expected to attenuate mucosal inflammation in chronic gastritis through α7nAChR-mediated suppression of NF-κB signaling and downregulation of TNF-α, IL-6, and IL-1β (Han et al., 2022; Hou et al., 2023; Matteoli and Boeckxstaens, 2016). Nevertheless, no dedicated clinical trial has yet evaluated taVNS in patients with active gastritis or peptic ulcer disease, and prudence dictates that such patients—particularly those with hypersecretory states, active ulceration, or uncontrolled Helicobacter pylori infection—should be enrolled cautiously in future studies, with acid-related symptoms and, where feasible, gastric pH or pepsin assays included as safety endpoints.

A parallel concern is whether enhancing vagal-cholinergic outflow could exacerbate diarrhea in IBS-D, where the dominant pathology is accelerated colonic transit and visceral hypersensitivity rather than slowed motility. The current clinical evidence base for taVNS in GIDs is heavily skewed toward IBS-C, with limited dedicated trials in IBS-D. In a recent IBS-C trial, intolerable diarrhea was reported as an adverse event in one patient receiving active taVNS during the early days of treatment, indicating that pro-motility effects can occasionally exceed the therapeutic window in susceptible individuals (Liu et al., 2024b). Conversely, a small open-label pilot study in mixed IBS and an adolescent percutaneous electrical nerve field stimulation trial reported symptom improvement and amelioration of both diarrhea and pain, attributed to dampened visceral hypersensitivity, anti-inflammatory effects, and rebalancing of autonomic tone rather than uniform acceleration of transit (Bonaz et al., 2017; Krasaelap et al., 2020). Mechanistic data also indicate that taVNS functions as a selective prokinetic: in healthy subjects, taVNS reduced gastric frequency while increasing peristaltic amplitude and normalized water-load-induced dysrhythmias toward physiological patterns rather than driving uniformly accelerated transit (Teckentrup et al., 2020; Steidel et al., 2021). Nevertheless, until adequately powered RCTs in IBS-D, functional diarrhea, and bile-acid diarrhea are completed, taVNS in these populations should be considered investigational. We suggest that future trials adopt subtype-specific stimulation protocols—for example, lower frequencies and intensities, or shorter daily durations in IBS-D—include stool-form and bowel-frequency diaries as safety endpoints, and incorporate stopping rules for symptom worsening, in line with the precision-medicine framework outlined in section 7.4.

## 8 Limitations of this review

This review paper still has certain limitations. First, the GIDs examined in this review are highly heterogeneous, encompassing FD, IBS, IBD, postoperative ileus, LPRD, PAP, and CP. While this demonstrates the wide applicability of taVNS across different gastrointestinal conditions, the distinct pathophysiological mechanisms underlying these disorders lead to diverse intervention responses. This variability complicates direct cross-study comparisons and the synthesis of coherent conclusions from disparate findings. Second, the number of high-quality clinical trials for each GID subtype remains limited, with notable heterogeneity in study designs, patient populations, stimulation protocols, and outcome measures. As a result, this review relies primarily on qualitative synthesis and narrative summary, which precludes the use of quantitative meta-analysis. This limitation restricts the ability to perform robust statistical evaluations of the overall efficacy of taVNS or to conduct meaningful subgroup analyses across different gastrointestinal conditions. Finally, although mechanistic insights from preclinical models have been summarized, much of the evidence linking taVNS to clinical outcomes remains correlational. Direct experimental validation of causal pathways in human studies is still lacking. Therefore, the proposed mechanisms of action require further consolidation through well-designed translational and clinical studies to establish stronger evidence for taVNS in gastrointestinal disorders.

## 9 Conclusion

taVNS as a non-invasive neuroregulation technique has demonstrated preliminary therapeutic potential in treating various GIDs, including FD and IBS-C. This is achieved through multiple mechanisms: modulating vagal tone, enhancing bidirectional brain-gut axis signaling, exerting cholinergic anti-inflammatory effects, and restoring gastrointestinal motility.

However, existing research faces significant limitations: the proposed mechanisms remain inadequately validated in humans; clinical studies commonly suffer from limited sample sizes, inconsistent stimulation parameters and protocols, lack of objective biomarker integration, and insufficient long-term efficacy evidence. Therefore, there is an urgent need to advance rigorously designed, large-scale, multicenter clinical trials. These trials should integrate multidimensional assessment systems, including neuroimaging and physiological monitoring, during treatment. Additionally, personalized stimulation protocols based on biomarkers and patient stratification should be developed to achieve reliable translation from mechanism exploration to clinically effective interventions.

## References

[B1] Akahoshi K. Oya M. Koga T. Shiratsuchi Y. (2018). Current clinical management of gastrointestinal stromal tumor. *World J. Gastroenterol.* 24 2806–2817. 10.3748/wjg.v24.i26.2806 30018476 PMC6048423

[B2] Austelle C. W. O’Leary G. H. Thompson S. Gruber E. Kahn A. Manett A. J. et al. (2022). A comprehensive review of vagus nerve stimulation for depression. *Neuromodulation* 25 309–315. 10.1111/ner.13528 35396067 PMC8898319

[B3] Badran B. W. Yu A. B. Adair D. Mappin G. DeVries W. H. Jenkins D. D. et al. (2019). Laboratory administration of transcutaneous auricular vagus nerve stimulation (taVNS): Technique, targeting, and considerations. *J. Vis. Exp. JoVE* 143. 10.3791/58984 30663712 PMC6867597

[B4] Bäez G. Vargas C. Arancibia M. Papuzinski C. Franco J. V. (2023). Non-chinese herbal medicines for functional dyspepsia. *Cochrane Database Syst. Rev.* 2023:CD013323. 10.1002/14651858.CD013323.pub2 37323050 PMC10267606

[B5] Bassotti G. Antonelli E. Villanacci V. Salemme M. Coppola M. Annese V. (2014). Gastrointestinal motility disorders in inflammatory bowel diseases. *World J. Gastroenterol.* 20 37–44. 10.3748/wjg.v20.i1.37 24415856 PMC3886030

[B6] Bedell A. Friedlander A. (2022). Management of sexual dysfunction in gastrointestinal disorders. *Gastroenterol. Clin. North Am.* 51 815–828. 10.1016/j.gtc.2022.06.012 36375998

[B7] Beyer G. Habtezion A. Werner J. Lerch M. M. Mayerle J. (2020). Chronic pancreatitis. *Lancet* 396 499–512. 10.1016/S0140-6736(20)31318-0 32798493

[B8] Bjarnason I. Scarpignato C. Holmgren E. Olszewski M. Rainsford K. D. Lanas A. (2018). Mechanisms of damage to the gastrointestinal tract from nonsteroidal anti-inflammatory drugs. *Gastroenterology* 154 500–514. 10.1053/j.gastro.2017.10.049 29221664

[B9] Black C. J. Drossman D. A. Talley N. J. Ruddy J. Ford A. C. (2020). Functional gastrointestinal disorders: Advances in understanding and management. *Lancet* 396 1664–1674. 10.1016/S0140-6736(20)32115-2 33049221

[B10] Black C. J. Paine P. A. Agrawal A. Aziz I. Eugenicos M. P. Houghton L. A. et al. (2022). British society of gastroenterology guidelines on the management of functional dyspepsia. *Gut* 71 1697–1723. 10.1136/gutjnl-2022-327737 35798375 PMC9380508

[B11] Bonaz B. Sinniger V. Pellissier S. (2017). Vagus nerve stimulation: A new promising therapeutic tool in inflammatory bowel disease. *J. Intern. Med.* 282 46–63. 10.1111/joim.12611 28421634

[B12] Browning K. N. Verheijden S. Boeckxstaens G. E. (2017). The vagus nerve in appetite regulation, mood, and intestinal inflammation. *Gastroenterology* 152 730–744. 10.1053/j.gastro.2016.10.046 27988382 PMC5337130

[B13] Bruner L. P. White A. M. Proksell S. (2023). Inflammatory bowel disease. *Primary Care* 50 411–427. 10.1016/j.pop.2023.03.009 37516511

[B14] Bu Y. Liang A. Hoffman B. U. Schiehser D. M. Case O. Simmons A. et al. (2026). A review of vagus nerve stimulation for disease: Comprehensive theory and evidence for mechanisms of action. *Comprehens. Physiol.* 16:e70109. 10.1002/cph4.70109 41781173 PMC12960021

[B15] Bucksot J. E. Morales Castelan K. Skipton S. K. Hays S. A. (2020). Parametric characterization of the rat hering-breuer reflex evoked with implanted and non-invasive vagus nerve stimulation. *Exp. Neurol.* 327:113220. 10.1016/j.expneurol.2020.113220 32027928 PMC7089831

[B16] Cai S. Li Q. Liu L. Zhao Q. Huang Q. Yuan K. (2025). Predictive value of acute neuroplastic response to taVNS in treatment outcome in persistent abdominal pain: A concurrent taVNS-EEG trial. *Brain Stimul.* 18 1511–1513. 10.1016/j.brs.2025.08.009 40812619

[B17] Campagnolo A. M. Priston J. Thoen R. H. Medeiros T. Assunção A. R. (2014). Laryngopharyngeal reflux: Diagnosis, treatment, and latest research. *Intern. Arch. Otorhinolaryngol.* 18 184–191. 10.1055/s-0033-1352504 25992088 PMC4297018

[B18] Capone F. Motolese F. Cruciani A. Rossi M. Musumeci G. Norata D. et al. (2025). The effects of transcutaneous auricular vagus nerve stimulation (taVNS) on cholinergic neural networks in humans: A neurophysiological study. *Clin. Neurophysiol.* 169 47–52. 10.1016/j.clinph.2024.11.012 39612592

[B19] Chen M. Ruan G. Chen L. Ying S. Li G. Xu F. et al. (2022). Neurotransmitter and intestinal interactions: Focus on the microbiota-gut-brain axis in irritable bowel syndrome. *Front. Endocrinol.* 13:817100. 10.3389/fendo.2022.817100 35250873 PMC8888441

[B20] Coffin B. Duboc H. (2022). Review article: Diagnostic and therapeutic approach to persistent abdominal pain beyond irritable bowel syndrome. *Alimentary Pharmacol. Therapeut.* 56 419–435. 10.1111/apt.17064 35656644

[B21] Cypess A. M. Cannon B. Nedergaard J. Kazak L. Chang D. C. Krakoff J. et al. (2025). Emerging debates and resolutions in brown adipose tissue research. *Cell Metab.* 37 12–33. 10.1016/j.cmet.2024.11.002 39644896 PMC11710994

[B22] de Araujo A. de Lartigue G. (2020). Non-canonical cholinergic anti-inflammatory pathway in IBD. *Nat. Rev. Gastroenterol. Hepatol.* 17 651–652. 10.1038/s41575-020-0356-y 32759984 PMC7826200

[B23] Décarie-Spain L. Hayes A. M. R. Lauer L. T. Kanoski S. E. (2024). The gut-brain axis and cognitive control: A role for the vagus nerve. *Sem. Cell Dev. Biol.* 156 201–209. 10.1016/j.semcdb.2023.02.004 36803834 PMC10427741

[B24] Di Rosa C. Altomare A. Terrigno V. Carbone F. Tack J. Cicala M. et al. (2023). Constipation-predominant irritable bowel syndrome (IBS-C): Effects of different nutritional patterns on intestinal dysbiosis and symptoms. *Nutrients* 15:1647. 10.3390/nu15071647 37049488 PMC10096616

[B25] Dong W. Wang Y. Zhang J. Lian H. Chen L. Peng T. et al. (2021). Transcutaneous auricular vagus nerve stimulation for functional dyspepsia: A randomized controlled trial. *World J. Acupuncture-Moxibustion* 31 165–171.

[B26] Drossman D. A. (2016). Functional gastrointestinal disorders: History, pathophysiology, clinical features and Rome IV. *Gastroenterology* 150 1262–1279.e2. 10.1053/j.gastro.2016.02.032 27144617

[B27] Du P. Maharjan A. Calder S. Schultz M. Schamberg G. Gharibans A. et al. (2024). Transcutaneous auricular vagus nerve stimulation normalizes induced gastric myoelectrical dysrhythmias in controls assessed by body-surface gastric mapping. *Neuromodulation* 27 333–342. 10.1016/j.neurom.2023.02.078 36997454

[B28] Duffy M. Boggiano V. L. Ganesh R. Mueller M. (2023). Functional gastrointestinal disorders. *Primary Care* 50 429–446. 10.1016/j.pop.2023.03.006 37516512

[B29] Esterita T. Dewi S. Suryatenggara F. G. Glenardi G. (2021). Association of functional dyspepsia with depression and anxiety: A systematic review. *J. Gastrointestinal Liver Dis. JGLD* 30 259–266. 10.15403/jgld-3325 33951117

[B30] Fan X. Wang Y. Cao J. Yu J. Tian J. Mi J. (2025). Clinical study status of diabetic gastrointestinal diseases. *Front. Endocrinol.* 16:1568552. 10.3389/fendo.2025.1568552 40270718 PMC12014455

[B31] Fikree A. Byrne P. (2021). Management of functional gastrointestinal disorders. *Clin. Med.* 21 44–52. 10.7861/clinmed.2020-0980 33479067 PMC7850201

[B32] Flynn S. Eisenstein S. (2019). Inflammatory bowel disease presentation and diagnosis. *Surg. Clin. North Am.* 99 1051–1062. 10.1016/j.suc.2019.08.001 31676047

[B33] Ford A. C. Mahadeva S. Carbone M. F. Lacy B. E. Talley N. J. (2020). Functional dyspepsia. *Lancet* 396 1689–1702. 10.1016/S0140-6736(20)30469-4 33049222

[B34] Ford A. C. Moayyedi P. Lacy B. E. Lembo A. J. Saito Y. A. Schiller L. R. et al. (2014). American college of gastroenterology monograph on the management of irritable bowel syndrome and chronic idiopathic constipation. *Am. J. Gastroenterol.* 109 (Suppl. 1), S2–S27. 10.1038/ajg.2014.187 25091148

[B35] Gaidos J. K. J. Hashash J. G. (2025). Monitoring inflammatory bowel disease activity: When, how, and why. *Am. J. Gastroenterol.* 120 1732–1741. 10.14309/ajg.0000000000003582 40488637

[B36] Giraudier M. Ventura-Bort C. Szeska C. Weymar M. (2025). A pooled analysis of the side effects of non-invasive transcutaneous auricular vagus nerve stimulation (taVNS). *Front. Hum. Neurosci.* 19:1539416. 10.3389/fnhum.2025.1539416 39981126 PMC11841445

[B37] Gracie D. J. Hamlin P. J. Ford A. C. (2019). The influence of the brain-gut axis in inflammatory bowel disease and possible implications for treatment. *Lancet Gastroenterol. Hepatol.* 4 632–642. 10.1016/S2468-1253(19)30089-5 31122802

[B38] Han J. Wei W. Wang H. C. Zhang T. Wang Y. Hou L. W. et al. (2022). Transcutaneous auricular vagus nerve stimulation promotes gastric motility by up-regulating α7nAChR and suppressing NF-κB p65 expression in duodenum in rats with functional dyspepsia. *Acupuncture Res.* 47 517–524. 35764519 10.13702/j.1000-0607.20220111

[B39] Hesampour F. Tshikudi D. M. Bernstein C. N. Ghia J. E. (2024). Exploring the efficacy of transcutaneous auricular vagus nerve stimulation (taVNS) in modulating local and systemic inflammation in experimental models of colitis. *Bioelectron. Med.* 10:29. 10.1186/s42234-024-00162-5 39648211 PMC11626753

[B40] Hijos-Mallada G. Sostres C. Gomollón F. (2022). NSAIDs, gastrointestinal toxicity and inflammatory bowel disease. AINE, toxicidad gastrointestinal y enfermedad inflamatoria intestinal. *Gastroenterol. Hepatol.* 45 215–222. 10.1016/j.gastrohep.2021.06.003 34157367

[B41] Hong G. S. Zillekens A. Schneiker B. Pantelis D. de Jonge W. J. Schaefer N. et al. (2019). Non-invasive transcutaneous auricular vagus nerve stimulation prevents postoperative ileus and endotoxemia in mice. *Neurogastroenterol. Motil.* 31:e13501. 10.1111/nmo.13501 30406957

[B42] Hou L. W. Fang J. L. Zhang J. L. Wang L. Wu D. Wang J. Y. et al. (2022). Auricular vagus nerve stimulation ameliorates functional dyspepsia with depressive-like behavior and inhibits the hypothalamus-pituitary-adrenal axis in a rat model. *Dig. Dis. Sci.* 67 4719–4731. 10.1007/s10620-021-07332-4 35064375

[B43] Hou L. W. Pei J. R. Wei W. Fang J. L. Wang D. Zhai W. H. et al. (2020). Effect and mechanism study on transcutaneous auricular vagus nerve stimulation for functional dyspepsia model rats. *World J. Acupuncture-Moxibust.* 30 49–56. 10.1016/j.wjam.2020.02.007

[B44] Hou L. W. Rong P. J. Li L. Wei W. Fang J. L. Zhang J. L. et al. (2021). Effects of transcutaneous auricular vagus nerve stimulation on autonomic nervous function in rats with functional dyspepsia. *Acupuncture Res.* 46 663–670. 34472751 10.13702/j.1000-0607.200965

[B45] Hou L. Rong P. Yang Y. Fang J. Wang J. Wang Y. et al. (2023). Auricular vagus nerve stimulation improves visceral hypersensitivity and gastric motility and depression-like behaviors via vago-vagal pathway in a rat model of functional dyspepsia. *Brain Sci.* 13:253. 10.3390/brainsci13020253 36831796 PMC9954117

[B46] Huang C. H. Wu C. H. Chan R. H. Chen C. H. Lin B. W. Lin C. K. (2025). Transcutaneous auricular vagus nerve stimulation accelerates postoperative ileus recovery by enhancing gastric motility complexity: A clinical study. *Brain Stimul.* 18 1091–1093. 10.1016/j.brs.2025.05.002 40404089

[B47] Huang Q. Li Q. He H. Zhao Q. Yuan K. Cai S. (2025). 30 Hz transcutaneous auricular vagus nerve stimulation alleviates abdominal pain by modulating EEG activity in the α frequency band of the brain. *CNS Neurosci. Therapeut.* 31:e70641. 10.1111/cns.70641 41169018 PMC12575443

[B48] Huang Y. Liu J. Lv C. Sun C. Meng M. Lowe S. et al. (2024). Integrative effects of transcutaneous auricular vagus nerve stimulation on esophageal motility and pharyngeal symptoms via vagal mechanisms in patients with laryngopharyngeal reflux disease. *Front. Neurosci.* 18:1287809. 10.3389/fnins.2024.1287809 38516311 PMC10954818

[B49] Hungin A. P. Whorwell P. J. Tack J. Mearin F. (2003). The prevalence, patterns and impact of irritable bowel syndrome: An international survey of 40,000 subjects. *Alimentary Pharmacol. Therapeut.* 17 643–650. 10.1046/j.1365-2036.2003.01456.x 12641512

[B50] Juel J. Brock C. Olesen S. S. Madzak A. Farmer A. D. Aziz Q. et al. (2017). Acute physiological and electrical accentuation of vagal tone has no effect on pain or gastrointestinal motility in chronic pancreatitis. *J. Pain Res.* 10 1347–1355. 10.2147/JPR.S133438 28615966 PMC5459955

[B51] Kaji N. Hori M. (2023). Interstitial cells of Cajal in gastrointestinal inflammatory diseases. *J. Smooth Muscle Res. Nihon Heikatsukin Gakkai Kikanshi* 59 1–13. 10.1540/jsmr.59.1 36792171 PMC9926098

[B52] Khasawneh M. Shaikh F. A. Ng C. E. Black C. J. Goodoory V. C. Ford A. C. (2024). Utility of irritable bowel syndrome subtypes and most troublesome symptom in predicting disease impact and burden. *Neurogastroenterol. Motil.* 36:e14756. 10.1111/nmo.14756 38321517

[B53] Kim A. Y. Marduy A. de Melo P. S. Gianlorenco A. C. Kim C. K. Choi H. et al. (2022). Safety of transcutaneous auricular vagus nerve stimulation (taVNS): A systematic review and meta-analysis. *Sci. Rep.* 12:22055. 10.1038/s41598-022-25864-1 36543841 PMC9772204

[B54] Kovacic K. Hainsworth K. Sood M. Chelimsky G. Unteutsch R. Nugent M. et al. (2017). Neurostimulation for abdominal pain-related functional gastrointestinal disorders in adolescents: A randomised, double-blind, sham-controlled trial. *Lancet Gastroenterol. Hepatol.* 2 727–737. 10.1016/S2468-1253(17)30253-4 28826627

[B55] Krasaelap A. Sood M. R. Li B. U. K. Unteutsch R. Yan K. Nugent M. et al. (2020). Efficacy of auricular neurostimulation in adolescents with irritable bowel syndrome in a randomized, double-blind trial. *Clin. Gastroenterol. Hepatol.* 18 1987–1994.e2. 10.1016/j.cgh.2019.10.012 31622740

[B56] Kreisberg E. Esmaeilpour Z. Adair D. Khadka N. Datta A. Badran B. W. et al. (2021). High-resolution computational modeling of the current flow in the outer ear during transcutaneous auricular vagus nerve stimulation (taVNS). *Brain Stimul.* 14 1419–1430. 10.1016/j.brs.2021.09.001 34517143 PMC8608747

[B57] Lee H. J. Wi S. Park S. Oh B. M. Seo H. G. Lee W. H. (2023). Exploratory investigation of the effects of tactile stimulation using air pressure at the auricular vagus nerve on heart rate variability. *Ann. Rehabil. Med.* 47 68–77. 10.5535/arm.22119 36599294 PMC10020049

[B58] Lembo A. Sultan S. Chang L. Heidelbaugh J. J. Smalley W. Verne G. N. (2022). AGA clinical practice guideline on the pharmacological management of irritable bowel syndrome with diarrhea. *Gastroenterology* 163 137–151. 10.1053/j.gastro.2022.04.017 35738725

[B59] Li J. Wang L. Zhang Y. Meng R. Zhu B. Chen J. D. Z. et al. (2025). Transcutaneous auricular vagal nerve stimulation improves functional dyspepsia with sleep disturbance via enhanced vagal activity: A randomized controlled trial. *Intern. J. Surg.* 112 961–972. 10.1097/JS9.0000000000003296 40905853 PMC12825726

[B60] Li S. Wang Y. Gao G. Guo X. Zhang Y. Zhang Z. et al. (2020). Transcutaneous auricular vagus nerve stimulation at 20 Hz improves depression-like behaviors and down-regulates the hyperactivity of HPA axis in chronic unpredictable mild stress model rats. *Front. Neurosci.* 14:680. 10.3389/fnins.2020.00680 32765210 PMC7378324

[B61] Liu C. H. Yang M. H. Zhang G. Z. Wang X. X. Li B. Li M. et al. (2020). Neural networks and the anti-inflammatory effect of transcutaneous auricular vagus nerve stimulation in depression. *J. Neuroinflamm.* 17:54. 10.1186/s12974-020-01732-5 32050990 PMC7017619

[B62] Liu J. Dai Q. Qu T. Ma J. Lv C. Wang H. et al. (2024a). Ameliorating effects of transcutaneous auricular vagus nerve stimulation on a mouse model of constipation-predominant irritable bowel syndrome. *Neurobiol. Dis.* 193:106440. 10.1016/j.nbd.2024.106440 38369213

[B63] Liu J. Lv C. Yin M. Zhu M. Wang B. Tian J. et al. (2024b). Efficacy and safety of transcutaneous auricular vagus nerve stimulation in patients with constipation-predominant irritable bowel syndrome: A single-center, single-blind, randomized controlled trial. *Am. J. Gastroenterol.* 120 2139–2153. 10.14309/ajg.0000000000003257 39689011 PMC12398348

[B64] Liu T. Wang Z. Li Y. Kang X. Wang X. Ren G. et al. (2025). Effects of transcutaneous auricular vagal nerve stimulation on chronic constipation: A multicenter, randomized controlled study. *United Eur. Gastroenterol. J.* 13 1550–1559. 10.1002/ueg2.70041 40359320 PMC12529053

[B65] Lynch C. J. Adams S. H. (2014). Branched-chain amino acids in metabolic signalling and insulin resistance. *Nat. Rev. Endocrinol.* 10 723–736. 10.1038/nrendo.2014.171 25287287 PMC4424797

[B66] Ma L. Wang H. B. Hashimoto K. (2025). The vagus nerve: An old but new player in brain-body communication. *Brain Behav. Immun.* 124 28–39. 10.1016/j.bbi.2024.11.023 39566667

[B67] Margolis K. G. Cryan J. F. Mayer E. A. (2021). The microbiota-gut-brain axis: From motility to mood. *Gastroenterology* 160 1486–1501. 10.1053/j.gastro.2020.10.066 33493503 PMC8634751

[B68] Matteoli G. Boeckxstaens G. E. (2016). The intestinal cholinergic anti-inflammatory pathway. *J. Physiol.* 594 5771–5780. 10.1113/JP271537 26959627 PMC5063935

[B69] Mena-Chamorro P. Espinoza-Palavicino T. Barramuño-Medina M. Romero-Arias T. Gálvez-García G. (2025). Impact of transcutaneous auricular vagus nerve stimulation (taVNS) on cognitive flexibility as a function of task complexity. *Front. Hum. Neurosci.* 19:1569472. 10.3389/fnhum.2025.1569472 40977857 PMC12445146

[B70] Mertz H. (2003). Review article: Visceral hypersensitivity. *Alimentary Pharmacol. Therapeut.* 17 623–633. 10.1046/j.1365-2036.2003.01447.x 12641510

[B71] Mishra P. Agrawal D. Artham P. (2020). Screening test for LPRD: History versus video laryngoscopy. *Indian J. Otolaryngol. Head Neck Surg.* 72 422–427. 10.1007/s12070-020-01828-7 33088769 PMC7544795

[B72] Mukhtar K. Nawaz H. Abid S. (2019). Functional gastrointestinal disorders and gut-brain axis: What does the future hold? *World J. Gastroenterol.* 25 552–566. 10.3748/wjg.v25.i5.552 30774271 PMC6371005

[B73] Naseri M. K. Hutson D. Grundy D. (1991). Vagal influences on gastric acid secretion in response to gastric distension in the ferret. *J. Autonomic Nervous Syst.* 36 25–31. 10.1016/0165-1838(91)90126-n 1753062

[B74] Noto T. Nagasaki M. Endo T. (1997). Role of vagus nerves and gastrin in the gastric phase of acid secretion in male anesthetized rats. *Am. J. Physiol.* 272(2 Pt 1), G335–G339. 10.1152/ajpgi.1997.272.2.G335 9124358

[B75] O’Brien S. J. Omer E. (2019). Chronic pancreatitis and nutrition therapy. *Nutrit. Clin. Pract.* 34 (Suppl. 1), S13–S26. 10.1002/ncp.10379 31535736

[B76] Park S. J. Yu Y. Zides C. G. Beyak M. J. (2022). Mechanisms of reduced leptin-mediated satiety signaling during obesity. *Intern. J. Obes.* 46 1212–1221. 10.1038/s41366-022-01079-2 35241786

[B77] Person H. Keefer L. (2021). Psychological comorbidity in gastrointestinal diseases: Update on the brain-gut-microbiome axis. *Prog. Neuro-Psychopharmacol. Biol. Psychiatry* 107:110209. 10.1016/j.pnpbp.2020.110209 33326819 PMC8382262

[B78] Ru O. Jin X. Qu L. Long D. Liu M. Cheng L. et al. (2023). Low-intensity transcutaneous auricular vagus nerve stimulation reduces postoperative ileus after laparoscopic radical resection of colorectal cancer: A randomized controlled trial. *Minerva Anestesiol.* 89 149–156. 10.23736/S0375-9393.22.16735-0 36326770

[B79] Sabo C. M. Grad S. Dumitrascu D. L. (2021). Chronic abdominal pain in general practice. *Dig. Dis.* 39 606–614. 10.1159/000515433 33631744

[B80] Sagui E. Claverie D. Bidaut W. Grelot L. (2024). Heart rate variability and cold-induced vascular dilation after stimulation of two different areas of the ear: A prospective, single-blinded, randomized crossover study. *BMC Compl. Med. Therap.* 24:83. 10.1186/s12906-024-04392-7 38350937 PMC10863191

[B81] Sahn B. Pascuma K. Kohn N. Tracey K. J. Markowitz J. F. (2023). Transcutaneous auricular vagus nerve stimulation attenuates inflammatory bowel disease in children: A proof-of-concept clinical trial. *Bioelectron. Med.* 9:23. 10.1186/s42234-023-00124-3 37849000 PMC10583463

[B82] Sairenji T. Collins K. L. Evans D. V. (2017). An update on inflammatory bowel disease. *Primary Care* 44 673–692. 10.1016/j.pop.2017.07.010 29132528

[B83] Sebastián Domingo J. J. (2022). Irritable bowel syndrome. Síndrome del intestino irritable. *Med. Clín.* 158 76–81. 10.1016/j.medcli.2021.04.029 34238582

[B84] Shi X. Hu Y. Zhang B. Li W. Chen J. D. Liu F. (2021). Ameliorating effects and mechanisms of transcutaneous auricular vagal nerve stimulation on abdominal pain and constipation. *JCI Insight* 6:e150052. 10.1172/jci.insight.150052 34138761 PMC8410029

[B85] Shi X. Zhao L. Luo H. Deng H. Wang X. Ren G. et al. (2024). Transcutaneous auricular vagal nerve stimulation is effective for the treatment of functional dyspepsia: A multicenter, randomized controlled study. *Am. J. Gastroenterol.* 119 521–531. 10.14309/ajg.0000000000002548 37787432

[B86] Simoni A. H. Ladebo L. Christrup L. L. Drewes A. M. Johnsen S. P. Olesen A. E. (2020). Chronic abdominal pain and persistent opioid use after bariatric surgery. *Scand. J. Pain* 20 239–251. 10.1515/sjpain-2019-0092 31756166

[B87] Sperber A. D. Bangdiwala S. I. Drossman D. A. Ghoshal U. C. Simren M. Tack J. et al. (2021). Worldwide prevalence and burden of functional gastrointestinal disorders, results of Rome foundation global study. *Gastroenterology* 160 99–114.e3. 10.1053/j.gastro.2020.04.014 32294476

[B88] Squeo F. Celiberto F. Ierardi E. Russo F. Riezzo G. D’Attoma B. et al. (2024). Opioid-induced constipation: Old and new concepts in diagnosis and treatment. *J. Neurogastroenterol. Motil.* 30 131–142. 10.5056/jnm23144 38576366 PMC10999847

[B89] Steidel K. Krause K. Menzler K. Strzelczyk A. Immisch I. Fuest S. et al. (2021). Transcutaneous auricular vagus nerve stimulation influences gastric motility: A randomized, double-blind trial in healthy individuals. *Brain Stimul.* 14 1126–1132. 10.1016/j.brs.2021.06.006 34187756

[B90] Teckentrup V. Neubert S. Santiago J. C. P. Hallschmid M. Walter M. Kroemer N. B. (2020). Non-invasive stimulation of vagal afferents reduces gastric frequency. *Brain Stimul.* 13 470–473. 10.1016/j.brs.2019.12.018 31884186

[B91] Valassi E. Scacchi M. Cavagnini F. (2008). Neuroendocrine control of food intake. *Nutrit. Metab. Cardiovas. Dis. NMCD* 18 158–168. 10.1016/j.numecd.2007.06.004 18061414

[B92] Wang W. Wang M. Ma C. Zhang Y. Li X. Wei Y. et al. (2025). Transcutaneous auricular vagus nerve stimulation attenuates stroke-heart syndrome: The role of parasympathetic activity. *Exp. Neurol.* 385:115094. 10.1016/j.expneurol.2024.115094 39637965

[B93] Wang Y. Duan C. Du X. Zhu Y. Wang L. Hu J. et al. (2025). Vagus nerve and gut-brain communication. *Neuroscientist* 31 262–278. 10.1177/10738584241259702 39041416

[B94] Wang Z. Stakenborg N. Boeckxstaens G. (2025). Postoperative ileus-Immune mechanisms and potential therapeutic interventions. *Neurogastroenterol. Motil.* 37:e14951. 10.1111/nmo.14951 39462772

[B95] Xin C. Li S. Feng B. Sun L. Wang Y. Zhang J. et al. (2025). Transcutaneous auricular vagus nerve stimulation promotes adipose tissue browning and mitochondrial integrity by regulating BCAA metabolism in obese rats: An experimental study. *Intern. J. Surg.* 111 4866–4871. 10.1097/JS9.0000000000002451 40387692

[B96] Yan L. Li H. Qian Y. Zhang J. Cong S. Zhang X. et al. (2024). Transcutaneous vagus nerve stimulation: A new strategy for Alzheimer’s disease intervention through the brain-gut-microbiota axis? *Front. Aging Neurosci.* 16:1334887. 10.3389/fnagi.2024.1334887 38476661 PMC10927744

[B97] Yang D. Forsmark C. E. (2017). Chronic pancreatitis. *Curr. Opin. Gastroenterol.* 33 396–403. 10.1097/MOG.0000000000000377 28771447

[B98] Yu Y. He X. Wang Y. Zhang J. Tang C. Rong P. (2022). Transcutaneous auricular vagal nerve stimulation inhibits limbic-regional P2X7R expression and reverses depressive-like behaviors in Zucker diabetic fatty rats. *Neurosci. Lett.* 775:136562. 10.1016/j.neulet.2022.136562 35245625

[B99] Yu Y. He X. Zhang J. Tang C. Rong P. (2021). Transcutaneous auricular vagal nerve stimulation inhibits hypothalamic P2Y1R expression and attenuates weight gain without decreasing food intake in Zucker diabetic fatty rats. *Sci. Prog.* 104:368504211009669. 10.1177/00368504211009669 33848220 PMC10358456

[B100] Yuan H. Silberstein S. D. (2016). Vagus nerve and vagus nerve stimulation, a comprehensive review: Part II. *Headache* 56 259–266. 10.1111/head.12650 26381725

[B101] Yue Y. Zou L. Li H. Xia Y. Ren Z. Yang F. et al. (2023). Therapeutic effect of implanted and non-invasive vagus nerve stimulation on heroin-induced anxiety. *Biochem. Biophys. Res. Commun.* 652 46–54. 10.1016/j.bbrc.2023.02.041 36809704

[B102] Zhang Q. Luo X. Wang X. H. Li J. Y. Qiu H. Yang D. D. (2024). Transcutaneous auricular vagus nerve stimulation for epilepsy. *Seizure* 119 84–91. 10.1016/j.seizure.2024.05.005 38820674

[B103] Zhang Y. Lu T. Dong Y. Chen Y. Chen J. D. Z. (2021a). Auricular vagal nerve stimulation enhances gastrointestinal motility and improves interstitial cells of Cajal in rats treated with loperamide. *Neurogastroenterol. Motil.* 33:e14163. 10.1111/nmo.14163 33991455

[B104] Zhang Y. Lu T. Meng Y. Maisiyiti A. Dong Y. Li S. et al. (2021b). Auricular vagal nerve stimulation improves constipation by enhancing colon motility via the central-vagal efferent pathway in opioid-induced constipated rats. *Neuromodulation* 24 1258–1268. 10.1111/ner.13406 33887080

[B105] Zhang Y. Song D. Liu R. Gong H. Wang C. Zhao M. G. et al. (2025). Transcutaneous auricular vagus nerve stimulation inhibits food intake and body weight gain through the orexin dependent pathway in high fat diet mice. *Sci. Rep.* 15:19286. 10.1038/s41598-025-01964-6 40456821 PMC12130493

[B106] Zhang Y. Zou N. Xin C. Wang Y. Zhang Z. Rong P. et al. (2024). Transcutaneous auricular vagal nerve stimulation modulates blood glucose in ZDF rats via intestinal melatonin receptors and melatonin secretion. *Front. Neurosci.* 18:1471387. 10.3389/fnins.2024.1471387 39564526 PMC11573758

[B107] Zhou J. Z. Chen H. Xu W. L. Fu Z. Zhou S. Zhu W. J. et al. (2024). Auricular vagal nerve stimulation inhibited central nerve growth factor/tropomyosin receptor kinase A/phospholipase C-gamma signaling pathway in functional dyspepsia model rats with gastric hypersensitivity. *Neuromodulation* 27 273–283. 10.1016/j.neurom.2023.01.007 36801128

[B108] Zhu Y. Xu F. Lu D. Rong P. Cheng J. Li M. et al. (2021). Transcutaneous auricular vagal nerve stimulation improves functional dyspepsia by enhancing vagal efferent activity. *Am. J. Physiol. Gastrointestinal Liver Physiol.* 320 G700–G711. 10.1152/ajpgi.00426.2020 33624527 PMC8887908

